# Development and validation of a nomogram for suicide attempts in patients with first-episode drug-naïve major depressive disorder

**DOI:** 10.3389/fpsyt.2024.1398733

**Published:** 2024-06-06

**Authors:** Junjun Liu, Ruixiang Tong, Zhaomin Lu, Zhiye Wang, Yangchun Wang, Yang Liu, Hsinsung Yuan, Fengnan Jia, Xiaobin Zhang, Zhe Li, Xiangdong Du, Xiangyang Zhang

**Affiliations:** ^1^ Nanjing Meishan Hospital, Nanjing, China; ^2^ Soochow University, Suzhou, China; ^3^ Suzhou Guangji Hospital, The Affiliated Guangji Hospital of Soochow University, Suzhou, China; ^4^ Chinese Academy of Sciences (CAS) Key Laboratory of Mental Health, Institute of Psychology, Chinese Academy of Sciences, Beijing, China

**Keywords:** validation, nomogram, predict, suicide attempts, major depressive disorder

## Abstract

**Objective:**

The risk of suicide can be decreased by accurately identifying high-risk suicide groups and implementing the right interventions. The aim of this study was to develop a nomogram for suicide attempts (SA) in patients with first-episode drug-naïve (FEDN) major depressive disorder (MDD).

**Methods:**

This study undertook a cross-sectional analysis of 1,718 patients diagnosed with FEDN MDD, providing comprehensive clinical data from September 2016 to December 2018. Data on anthropometric and sociodemographic factors were gathered, and the severity of depression and anxiety was evaluated using the 17-item Hamilton Depression Scale (HAMD-17) and the Hamilton Anxiety Scale (HAMA), respectively. Additionally, thyroid hormone levels, lipid profile parameters, and fasting blood glucose (FBG) were measured. Suicide attempt (SA) history was verified based on an amalgamation of medical records, patient interviews, and family interviews. Participants were randomly divided into a training group (70%, n = 1,204) and a validation group (30%, n = 514). In the training group, LASSO analysis and multivariate regression were used to identify variables associated with SA. A nomogram was then constructed using the identified risk factors to estimate the likelihood of SA within the training group. To assess the accuracy, the area under the receiver operating characteristic curve (AUC) was utilized, and calibration plots were employed to evaluate calibration. Additionally, decision curve analysis (DCA) was performed to assess the precision of the model. Finally, internal validation was carried out using the validation group.

**Results:**

A practical nomogram has been successfully constructed, incorporating HAMD, HAMA, thyroid stimulating hormone (TSH), thyroid peroxidase antibody (TPOAb), and systolic blood pressure (SBP) parameters, to estimate the probability of SA in Chinese patients diagnosed with FEDN MDD. The pooled area under the ROC for SA risk in both the training and validation groups was found to be 0.802 (95% CI: 0.771 to 0.832) and 0.821 (95% CI: 0.774 to 0.868), respectively. Calibration analysis revealed a satisfactory correlation between the nomogram probabilities and the actual observed probabilities. The clinical applicability of the nomogram was confirmed through decision curve analysis. To enhance accessibility for clinicians and researchers, an online version of the nomogram can be accessed at https://doctorjunjunliu.shinyapps.io/dynnomapp/.

**Conclusions:**

We constructed and validated a nomogram for the early detection of FEDN MDD patients with a high risk of SA, thereby contributing to the implementation of effective suicide prevention programs.

## 1 Introduction

Major depressive disorder (MDD), a pervasive psychiatric condition characterized by persistent low mood, anhedonia, fatigue, sleep disturbances, and recurrent thoughts of death or suicidal ideation, stands as one of the most prevalent mental health disorders worldwide (1). According to the World Health Organization’s 2017 report, the global prevalence of depression escalated by 18.4% from 2005 to 2015, affecting approximately 322 million individuals, or 4.4% of the world’s population (2). A systematic analysis conducted in China revealed that the lifetime, 12-month, and current prevalence rates of MDD were 3.3%, 2.3%, and 1.6%, respectively (3). The direst and most pressing consequence of MDD manifests itself in the form of suicide, with a staggering 15% of recurrent MDD patients resorting to this tragic act even after receiving pharmacological treatment (4). Suicide has emerged as a consequential public health challenge both in China and worldwide. Moreover, suicide attempts (SA) surpass suicide deaths in prevalence and serve as robust indicators of suicide mortality (5). A history of depression is associated with a 30-fold increase in suicide risk, while individuals with a prior SA history face an even greater risk of suicide (6). Considering that suicide attempt history stands as the primary prognosticator of suicide mortality, attending to the care of suicide attempt survivors emerges as a paramount concern in the realm of suicide prevention (7).

The multifaceted nature of SA encompasses a range of biological, psychological, and environmental factors (8), adding to its intricate nature. In the pursuit of advancing clinical suicide risk assessment, scientists are investigating the neurobiological mechanisms underlying suicidal behavior with the objective of identifying novel biomarkers. This endeavor stems from the existing scarcity of dependable biomarkers in this domain. The association between thyroid dysfunction and depression has long been established, with some experts postulating that thyroid hormones may be involved in the underlying neurobiological mechanisms of suicidal behavior (9). Additionally, several studies have reported a correlation between lipid abnormalities and suicidality (10). Notably, research findings indicate that individuals with MDD and lower total cholesterol (TC) levels exhibit a higher prevalence of SA (11).

Previous research has demonstrated that SA in individuals experiencing depression is linked with adverse outcomes such as more severe medical conditions, inadequate treatment responses, and even suicide itself. However, it has come as a surprise that the diagnostic rate of major depression with SA was low (12). This discrepancy could potentially be attributed to the influence of social expectation bias on survey inquiries assessing SA, compounded by the sensitivity of the subject matter, which may prompt individuals to respond negatively (13). Consequently, there exists the possibility that the actual incidence of SA within the studied population is underestimated. Furthermore, an investigation into suicide stigma has uncovered prejudiced attitudes and discriminatory practices towards those who have attempted or died by suicide (14). To accurately ascertain the prevalence of SA among individuals with MDD, it is imperative to employ instruments that eradicate social desirability bias or appraise response verity. By doing so, the development of effective strategies for suicide prevention can be facilitated.

Nomograms hold substantial potential for guiding clinical decision-making for patient management (15). Accurate identification of SA among individuals with depression is of paramount importance. However, this matter has only been comprehensively addressed in two studies (16, 17), where the examined factors were not exhaustive, neglecting significant components such as metabolic parameters and thyroid hormones, among others. Moreover, the sample sizes in these investigations were relatively small, consisting of 474 and 273 cases, respectively. Previous research has revealed that the rate of SA was 21 times higher during the acute phase and 4 times higher during the partial remission period compared to the full remission period in MDD patients (18). Given the imperative of reducing suicide fatalities through early detection, the identification of risk factors associated with SA in individuals with MDD, particularly during the acute phase, assumes critical significance.

To the best of our knowledge, few studies have developed a nomogram to determine the probability of SA in patients with first-episode drug-naïve (FEND) MDD, particularly within the Chinese population. Patients with FEND MDD offer a unique opportunity to study the connection between SA and associated risk factors in MDD patients without the confounding influence of medications and lifestyle interventions, such as disease course and comorbid medical illness (13). Therefore, the objective of this study was to construct and validate a novel nomogram combining evidence-based and statistically significant clinical features to identify the risk of SA in a relatively large sample size of patients with FEND MDD in China.

## 2 Materials and methods

### 2.1 Subjects

This cross-sectional study was carried out at the First Hospital of Shanxi Medical University in Taiyuan, Shanxi Province, China, between September 2016 and December 2018. A total of 1718 FEDN MDD patients were enrolled, comprising 588 males and 1130 females. Two qualified psychiatrists diagnosed MDD using the Structured Clinical Interview for the Diagnostic and Statistical Manual of Mental Disorders, Fourth Edition (SCID). The inclusion criteria consisted of: (1) individuals of Han Chinese ethnicity; (2) age between 18 and 60 years; (3) fulfillment of the Diagnostic and Statistical Manual of Mental Disorders (DSM) IV-TR criteria for MDD; (4) currently experiencing an acute first episode of depressive symptoms; (5) no previous exposure to antidepressants or antipsychotics; and (6) a 17-item Hamilton Depression Rating Scale (HAMD) score of ≥ 24. The exclusion criteria were as follows: (1) presence of a severe physical illness such as cancer, persistent infection, epilepsy, brain injury, and stroke (n = 9); (2) being pregnant or lactating women (n = 10); (3) alcohol or substance abuse, excluding smoking (n = 9); and (4) meeting any other major Axis I disorders (n = 15); (5) difficulty in conducting the interview (n = 5); and (6) refusal to participate in the study (n = 21).

The present study meticulously followed the Transparent Reporting of a Multivariable Prediction Model for Individual Prognosis or Diagnosis (TRIPOD) guidelines, ensuring comprehensive reporting of studies. Ethical approval for this study was granted by the Institutional Review Board of the First Hospital, Shanxi Medical University (No. 2016-Y27), and all participants provided written informed consent prior to their involvement. Within the context of this study, FEDN patients were operationally defined as those experiencing their first episode of symptoms without any previous exposure to antidepressants or antipsychotics (12).

### 2.2 Sociodemographic characteristics and anthropometric data

Sociodemographic characteristics and general information regarding the patients, including age (in years), gender (male, female), marital status (single, married), education level (junior high school, senior high school, college, postgraduate), age of symptom onset (in years), and duration of illness (in months), were collected through the utilization of a meticulously designed, structured questionnaire.

Trained professionals with meticulous attention to detail employed highly accurate measurement techniques to determine weight and height, ensuring an accuracy level within 1 cm and 1 kg, respectively. Body weight measurements were conducted with subjects donning lightweight clothing. The body mass index (BMI) was subsequently calculated by dividing the weight in kilograms by the square of the height in meters (kg/m^2^). To measure blood pressure, researchers adhered to established protocols and utilized calibrated equipment (model HBP-9020; Omron Corp., Kyoto, Japan) to record readings from the right arm of participants, following a resting period of at least five minutes. The mean values of the two measurements were used to calculate systolic blood pressure (SBP) and diastolic blood pressure (DBP) for further analysis.

Information was collected through face-to-face interviews to investigate participants’ histories of SA. SA was defined as self-harming behavior with the intention to end one’s life. The question “Have you ever attempted suicide?” was taken from the WHO/EURO multicenter study (19). Those who answered “yes” were considered to have attempted suicide. Further information about the frequency, method, and exact dates of SA was obtained. If answers were unclear, family members, relatives, or friends were interviewed. Suicide attempts included one patient with four attempts, two patients with three attempts, 26 patients with two attempts, and 317 patients with one attempt. The methods of suicide included jumping from a height, cutting one’s wrists, crashing into a car, hanging oneself, using gas, burning charcoal, and throwing oneself into a river. A total of 346 MDD patients reported SA in the past month. Accordingly, subjects were divided into two groups: suicide attempters (SA = 346) and non-suicide attempters (NSA = 1372).

### 2.3 Clinical assessment

The Hamilton Depression Scale (HAMD-17), consisting of 17 items, was used to assess the severity of depression. Among these components, eight items were appraised on a 5-point scale ranging from 0 (absent) to 4 (severe), while the remaining nine items employed a scale ranging from 0 (absent) to 2 (symptom-specific severity description). Higher scores, ranging from 0 to 52, indicate more severe depressive symptoms. Patients in this study had to achieve a minimum score of 24 to be included. Notably, this scale has been widely used in China and has previously demonstrated strong reliability and validity (20).

For the assessment of anxiety symptoms, the Hamilton Anxiety Rating Scale (HAMA) in its Chinese version was employed (21). This scale captured both somatic manifestations, encompassing physical discomfort associated with anxiety, and psychogenic aspects, including mental restlessness and psychological distress. Comprising 14 items reflective of specific symptoms, the HAMA was scored on a 5-point Likert scale ranging from 0 (absent) to 4 (severe symptoms). The overall score ranged from 0 to 56, signifying the severity of anxiety experienced by participants.

Two psychiatrists, each with over five years of clinical experience, underwent pre-study training on the utilization of HAMA and HAMD. Subsequent assessments conducted after the training demonstrated that interobserver correlation coefficients for total scores on HAMA and HAMD consistently exceeded 0.8. The raters were unaware of the patients’ clinical conditions during the assessments.

### 2.4 Blood samples

All participants underwent an overnight fasting period prior to the collection of blood samples, which occurred between 6:00 and 9:00 a.m. The samples were promptly transported to the hospital’s laboratory facility and analyzed utilizing the Abbott Immulite 2000 SR clinical analyzer by 11:00 a.m. on the same day. Measurements included thyroid stimulating hormone (TSH), antithyroglobulin (TgAb), thyroid peroxidase antibody (TPOAb), free triiodothyronine (FT3), and free thyroxine (FT4). Additionally, the lipid profile, encompassing low-density lipoprotein cholesterol (LDL-C), high-density lipoprotein cholesterol (HDL-C), total cholesterol (TC), triglycerides (TG), as well as fasting blood glucose (FBG), was quantified utilizing the Abbott ARCHITECT c8000 auto-analyzer.

### 2.5 Statistical analysis

All analyses were performed using SPSS 22.0, R software (version 4.1.0; http://www.r-project.org) and EmpowerStats (http://www.empowerstats.com, X&Y Solution, Inc., Boston, Massachusetts, USA). To allocate patients into training and validation groups, a random number generator approach available in SPSS software was employed. Specifically, 70% (1,204 individuals) of the total recruited participants were randomly assigned to the training group, while the remaining 30% (514 individuals) were assigned to the validation group. The normality of continuous variables’ distributions was assessed using the Kolmogorov-Smirnov test, and logarithmic transformations were applied to non-normally distributed continuous variables (TGAb and TPOAb). Categorical variables were presented as counts (percentages), whereas continuous variables were reported as the mean (standard deviation). Demographic and clinical characteristics between the training and validation groups were compared using t-tests and Chi-Square tests based on the nature of the variables.

The nomogram construction consisted of three steps. Initially, we utilized univariate and stepwise forward multivariate logistic regression analyses to identify factors associated with SA in the training group. The collinearity of the variables included in the regression equation was assessed by employing the variance inflation factor (VIF). A VIF exceeding 5 indicates the presence of multicollinearity. In the subsequent step, regression analyses utilizing the least absolute shrinkage and selection operator (LASSO) were performed on the training group to identify relevant risk factors through shrinkage and variable selection. The LASSO regression method eliminates irrelevant variables, which are expected to possess a negligible coefficient, in order to derive a subset of related variables. The parameter for SA is defined as a binary variable, denoting either true or false with respect to the dependent variable. LASSO regression analysis was executed based on the binomial SA and the log-likelihood measure of Type II error, employing tenfold cross-validation to standardize and normalize the included variables before determining the optimal lambda value. Lambda.lse yielded a model with the lowest number of independent variables exhibiting satisfactory performance. Only the features shared by both models were selected to construct the nomogram, which was visualized via a web-based interface. This resource offers clinicians an intuitive and quantitative instrument to identify patients at highest risk of SA. Subsequently, we assessed the precision of the risk model using data from the training and validation sets, employing diverse validation approaches. The ability of the risk nomogram to discriminate between true and false positives was evaluated using the area under the receiver operating characteristic (ROC) curve. To evaluate the suicide risk using the nomogram, calibration curves and the Hosmer-Lemeshow test (HL test) were utilized. The clinical relevance of the nomogram was further assessed using decision curve analysis (DCA), based on the net benefit at varying threshold probabilities. Statistical significance was defined as a p-value less than 0.05.

## 3 Results

### 3.1 Baseline characteristics of the study

**Figure 1:**
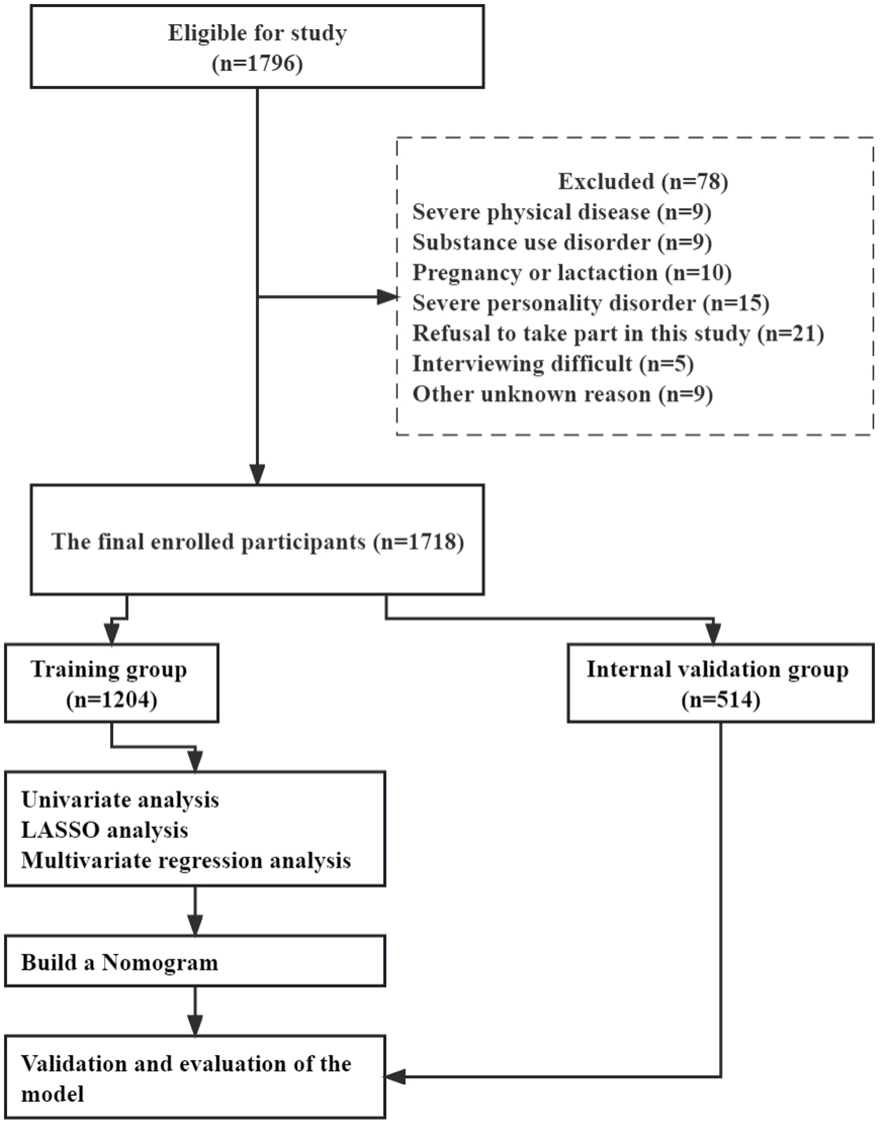
Flow chart of this study.

The study population included 1718 patients with FEDN MDD after applying eligibility criteria and excluding participants. **Figure 1** provides a flowchart of the study design process. The training dataset consisted of 1204 patients, while the validation dataset included 514 patients. These patients were randomly assigned to either the training or validation set using a 7:3 ratio. The eligible participants had a male gender ratio of 34.23% (588/1718) and a mean age of 34.87 ± 12.43 years. Twenty-one percent of the patients (346/1718) had SA. The incidence of SA was 244 (20.27%) and 102 (19.84%) in the training and validation groups, respectively. No significant differences were observed in sociodemographic, physical exam, or laboratory characteristics between the training and validation groups (**Table 1**).

**Table 1:** Characteristics of the training set and validation set.

Variables	Total (n=1,718)	Training group (n=1204)	Validation group (n=514)	P-value
Age (years)	34.87 ± 12.43	35.09 ± 12.35	34.35 ± 12.62	0.257
Duration of illness (months)	5.00 (3.00–8.00)	5.00 (3.00–8.00)	5.00 (3.00–8.00)	0.550
Age at onset (years)	34.66 ± 12.31	34.88 ± 12.22	34.14 ± 12.51	0.254
HAMD	30.30 ± 2.94	30.38 ± 2.98	30.10 ± 2.83	0.069
HAMA	20.80 ± 3.47	20.89 ± 3.47	20.58 ± 3.47	0.096
TSH (uIU/ml)	5.07 ± 2.56	5.11 ± 2.51	4.98 ± 2.68	0.319
Log (TGAb) (IU/l)	1.50 ± 0.50	1.51 ± 0.51	1.49 ± 0.49	0.479
Log (TPOAb) (IU/l)	1.41 ± 0.51	1.42 ± 0.52	1.40± 0.50	0.314
FT3 (pmol/l)	4.90 ± 0.72	4.91 ± 0.72	4.88 ± 0.73	0.387
FT4 (pmol/l)	16.70 ± 3.10	16.72 ± 3.03	16.66 ± 3.24	0.676
FBG (mmol/l)	5.40 ± 0.65	5.40 ± 0.65	5.39 ± 0.64	0.636
TC (mmol/l)	5.25 ± 1.11	5.26 ± 1.11	5.22 ± 1.10	0.446
HDL-c (mmol/l)	1.22 ± 0.29	1.22 ± 0.29	1.22 ± 0.29	0.930
TG (mmol/l)	2.17 ± 0.99	2.18 ± 0.99	2.14 ± 0.98	0.446
LDL-c (mmol/l)	2.98 ± 0.86	2.99 ± 0.86	2.98 ± 0.87	0.823
BMI (kg/m^2^)	24.37 ± 1.92	24.40 ± 1.88	24.30 ± 2.02	0.356
Systolic pressure (mmHg)	119.48 ± 10.91	119.75 ± 10.84	118.85 ± 11.07	0.119
Diastolic pressure (mmHg)	75.95 ± 6.74	76.05 ± 6.73	75.72 ± 6.78	0.353
Gender				0.094
Male	588 (34.23%)	397 (32.97%)	191 (37.16%)	
Female	1130 (65.77%)	807 (67.03%)	323 (62.84%)	
Education				0.486
Junior high school	413 (24.04%)	280 (23.26%)	133 (25.88%)	
Senior high school	760 (44.24%)	532 (44.19%)	228 (44.36%)	
College	449 (26.14%)	320 (26.58%)	129 (25.10%)	
Postgraduate	96 (5.59%)	72 (5.98%)	24 (4.67%)	
Marital status				0.307
Single	502 (29.22%)	343 (28.49%)	159 (30.93%)	
Marriage	1216 (70.78%)	861 (71.51%)	355 (69.07%)	
Suicide attempts				0.842
No	1372 (79.86%)	960 (79.73%)	412 (80.16%)	
Yes	346 (20.14%)	244 (20.27%)	102 (19.84%)	

### 3.2 Characteristics of selection by LASSO regression analysis

**Figure 2:**
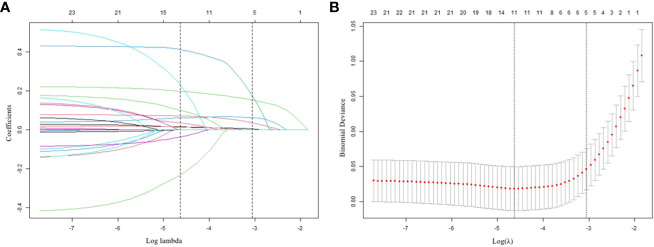
Results of the LASSO regression analysis **(A)** Plot of the LASSO coefficient profiles. **(B)** tuning parameter (l) selection cross-validation.

**Table 1** encompasses an extensive array of 21 potential demographic and clinical characteristics that could serve as risk factors. These factors include age, gender, education, marital status, duration of illness, age at onset, SBP, DBP, FT3, FT4, TSH, Log (TGAb), Log (TPOAb), TG, TC, HDL-c, LDL-c, FBG, BMI, HAMD, and HAMA. To identify the most significant variables among this extensive set, LASSO regression analysis, accompanied by 10-fold cross-validation, was performed. The LASSO method is a statistical technique used for variable selection in regression models. It is particularly useful when there are a large number of predictors, and it is necessary to identify the most relevant variables while discarding any confounding variables. The LASSO achieves this by minimizing the coefficients of less relevant variables to zero, effectively excluding them from the model (22). This robust approach effectively selects statistically meaningful variables, resulting in the reduction of the initial 21 variables to a concise set of five: HAMD, HAMA, TSH, Log (TPOAb), and SBP (**Figures 2A, B**). The specific coefficients for each variable are diligently presented in **Table 2**, with the associated Lambda.lse value determined to be 0.0473.

**Table 2:** Coefficients and lambda.1se value of the LASSO regression based on the training cohort.

Factors	Coefficients	Lambda.lse
HAMD	0.037	0.0473
HAMA	0.154	
TSH (uIU/ml)	0.062	
Log (TPOAb) (IU/l)	0.174	
SBP (mmHg)	0.004	

### 3.3 Univariate and multivariate logistic regression analysis in the training group

**Table 3** presents the results of univariate and multivariate logistic regression analyses conducted on 1204 Chinese patients with FEDN MDD in the training group. Forward stepwise regression was used to construct the best training group model, which identified HAMD, HAMA, TSH, Log (TPOAb), BMI, and SBP as possible risk variables for newly diagnosed SA. A table detailing suicide attempts and related factors by multivariable logistic regression in all patients has been added as **Supplementary Table 1**.

**Table 3:** Risk factors for suicide attempts according to univariate and multivariate Logistic regression model in the training group.

Variables	Univariate analysis		Multivariate analysis	
	OR (95%CI)	P	OR (95%CI)	P
Age (years)	1.01 (1.00, 1.02)	0.079		
Log (Duration of illness) (months)	1.80 (1.17, 2.78)	0.008		
Age at onset (years)	1.01 (1.00, 1.02)	0.078		
HAMD	1.35 (1.28, 1.43)	<0.001	1.10 (1.02,1.18)	0.009
HAMA	1.35 (1.29, 1.42)	<0.001	1.24 (1.17,1.31)	<0.001
TSH (uIU/ml)	1.35 (1.27, 1.44)	<0.001	1.09 (1.01,1.17)	0.031
Log (TGAb) (IU/l)	2.32 (1.81, 2.98)	<0.001		
Log (TPOAb) (IU/l)	2.62 (2.04, 3.36)	<0.001	1.78 (1.33,2.39)	<0.001
FT3 (pmol/l)	1.00 (0.83, 1.22)	0.968		
FT4 (pmol/l)	1.00 (0.96, 1.05)	0.840		
FBG (mmol/l)	1.71 (1.38, 2.11)	<0.001		
TC (mmol/l)	1.68 (1.47, 1.92)	<0.001		
HDL-c (mmol/l)	0.26 (0.15, 0.42)	<0.001		
TG (mmol/l)	1.14 (0.99, 1.31)	0.064		
LDL-c (mmol/l)	1.44 (1.22, 1.70)	<0.001		
BMI (kg/m^2^)	0.98 (0.91, 1.06)	0.633	0.92 (0.84,0.998)	0.045
Systolic pressure (mmHg)	1.06 (1.04, 1.07)	<0.001	1.03 (1.01,1.05)	0.001
Diastolic pressure (mmHg)	1.08 (1.06, 1.10)	<0.001		
Gender				
Male	1.0			
Female	1.19 (0.88, 1.62)	0.256		
Education				
Junior high school	1.0			
Senior high school	0.74 (0.52, 1.05)	0.096		
College	0.75 (0.50, 1.11)	0.149		
Postgraduate	1.16 (0.64, 2.10)	0.619		
Marital status				
Single	1.0			
Marriage	1.18 (0.86, 1.63)	0.301		

### 3.4 Development of the individualized nomogram

**Figure 3:**
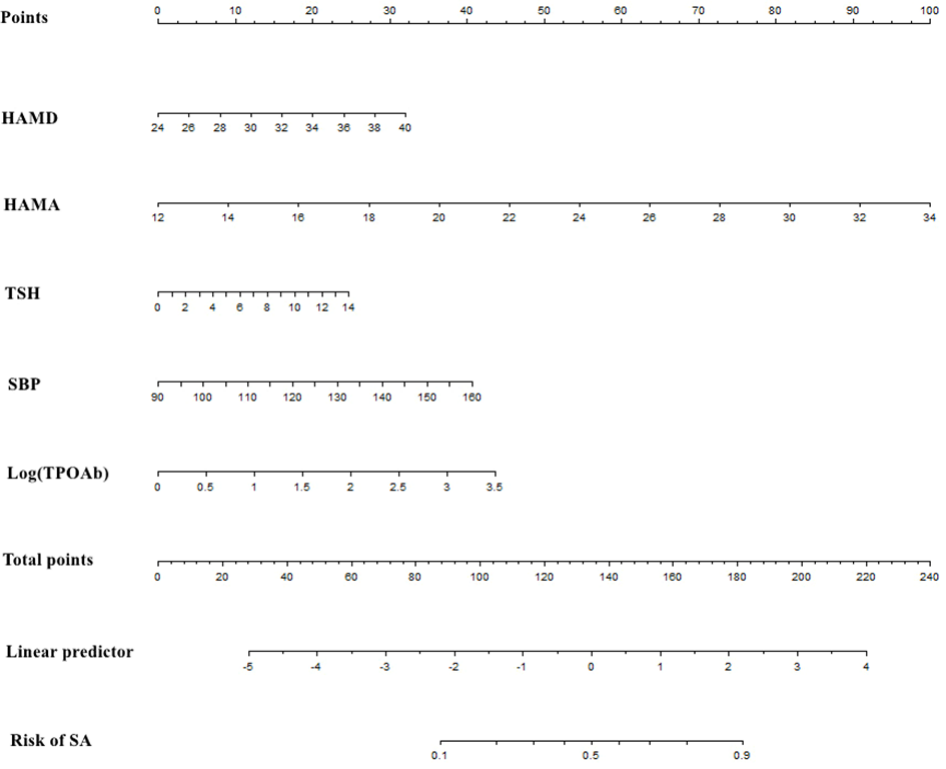
Nomogram to evaluate SA in FEDN MDD patients.

The overlapping region between the multivariate model and LASSO regression was used to select relevant clinical features. Pertinent to the discoveries derived from these models, a nomogram was constructed to estimate the individual risk of SA in Chinese patients with FEDN MDD. The nomogram consists of five critical risk factors: HAMD, HAMA, TSH, Log (TPOAb), and SBP. It is a practical and quantitative tool, as seen in **Figure 3**. Demonstrating its practicality and quantitative nature, this nomogram is visually presented in **Figure 3**. Evaluating each factor on the point scale, an individual’s risk can be determined by summing the assigned points, ultimately reflecting the overall risk of SA.

### 3.5 Performance of the nomogram

**Figure 4:**
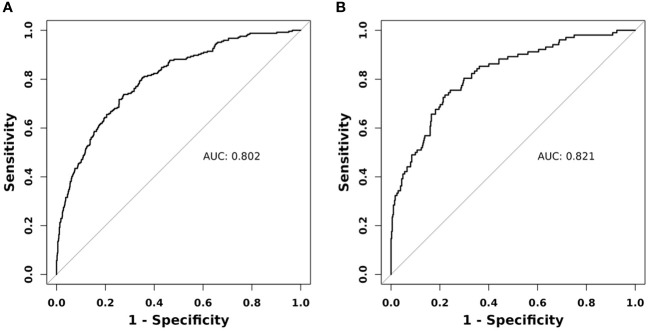
ROC curve of the nomogram in the **(A)** training group and **(B)** validation group.

The effectiveness of the nomogram was assessed through the utilization of the receiver operating characteristic (ROC) curve. In the training group, the ROC curve exhibited an area under the curve (AUC) of 0.802, with a 95% confidence interval (CI) ranging from 0.771 to 0.832. Notably, the model displayed a sensitivity of 73.77% and a specificity of 72.50%, as depicted in **Figure 4A**. Similarly, the ROC curve for the validation group, as illustrated in **Figure 4B**, demonstrated an AUC of 0.821, accompanied by a 95% CI spanning from 0.774 to 0.868. The sensitivity and specificity values for the validation group were determined to be 73.53% and 77.91%, respectively.

**Figure 5:**
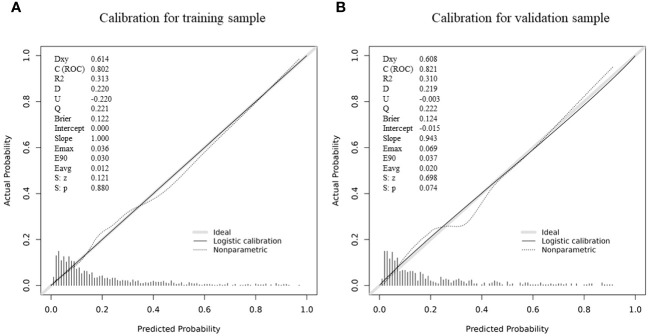
Calibration plots of the nomogram in the **(A)** training group and **(B)** validation group.

The calibration of the nomogram was assessed using the calibration curve and the Hosmer-Lemeshow test. Remarkably, both the training and validation cohorts exhibited excellent conformity to the model, as illustrated by the calibration curves presented in **Figures 5A, B**. Furthermore, the Hosmer-Lemeshow test, a robust statistical measure, demonstrated no discernible divergence between the projected risk of SA and the actual observed risk (P > 0.05).

**Figure 6:**
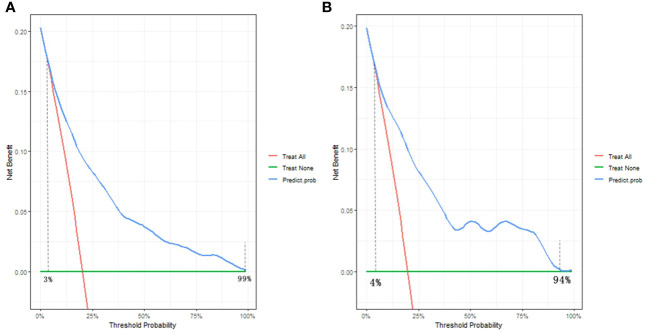
Decision curve analysis of the nomogram in the **(A)** training group and **(B)** validation group. The horizontal and vertical axes represent the threshold probability and net benefit, respectively. The lines between the horizontal axis and vertical axis display the benefit of divergence.

The decision curve analysis (DCA) findings, depicted in **Figures 6A, B**, offer a comprehensive assessment of the clinical utility of the nomogram in both the training and validation groups. Along the horizontal and vertical axes, the figures illustrate the threshold probability and net benefit, respectively. The lines connecting the axes effectively represent the benefits associated with different related variables. Through the DCA curves, it is evident that employing this nomogram for identification of the risk of SA in the present study confers greater advantages when the threshold probability ranges between 3% and 99%. Moreover, the DCA demonstrates that the threshold probabilities for the validation set fall within a range of 4% to 94%. These DCA results provide valuable insights into the optimal utilization of the nomogram in assessing SA risk.

### 3.6 Development of a webserver for easy access to our new model

For enhanced accessibility and improved usability, our nomogram has been made available online at https://doctorjunjunliu.shinyapps.io/dynnomapp/. This web-based resource serves as a valuable tool for both researchers and clinicians, facilitating a more streamlined and efficient utilization of the nomogram. By inputting relevant clinical variables and analyzing the resultant data and tables generated through the web server, users can easily calculate the anticipated incidence of SA in individuals with FEDN MDD. For instance, if a patient has a HAMD score of 36, a HAMA score of 30, a TSH level of 8uIU/ml, a SBP value of 125mmHg, and a Log (TPOAb) level of 2IU/l, the probability of SA risk would be calculated as 0.807. This probability implies that the patient has an 80.7% chance of experiencing SA.

## 4 Discussion

In this cross-sectional study, we have developed and validated a nomogram model that incorporates sociodemographic characteristics, anthropometric data, clinical laboratory parameters, and psychological assessment scale indicators. The purpose of this model is to identify the risk of SA in the acute phase among Chinese patients with FEDN MDD. By utilizing this nomogram, clinicians can effectively identify individuals who are at higher risk for SA. The components of the nomogram include HAMD, HAMA, TSH, Log (TPOAb), and SBP. Notably, the nomogram demonstrates excellent performance and consistent calibration curve accuracy in both the training and validation groups. Furthermore, decision curve analysis reveals the clinical relevance of this nomogram. The objective and easily accessible properties of our nomogram contribute to its significance as a practical tool.

There exists a substantial body of evidence supporting the association between increased severity of depression and the likelihood of suicidal behavior, including SA. It has been found that depression strongly influences the occurrence of SA. In a 2-year follow-up study, it was observed that depression raised the risk of SA sevenfold, whereas each prior suicide attempt increased the risk by 30% (23). Preclinical and clinical evidence suggests that dysregulation of central serotonergic (5-HT) neurotransmission is linked to depression, suicidal behavior, and aggressive acts towards oneself (24). Most currently used antidepressant medications exert their therapeutic effects by acting on 5-HTergic neurons, thus highlighting the importance of the brain’s 5-HT system as a target for antidepressant treatment (25). Pharmacological evidence strongly supports the role of 5-HT1A receptors in the mechanisms underlying depression and depressive-like behaviors, as exemplified by the antidepressant effects of 5-HT1A receptor agonists that are comparable to those of traditional antidepressants from the selective serotonin reuptake inhibitor (SSRI) family (26–29). Studies have reported higher 5-HT1A binding potentials for 5-HT1A autoreceptors in the raphe nuclei of depressed individuals who have attempted suicide, as well as in suicide victims (30, 31). Furthermore, reduced activity of downstream effectors of cortical 5-HT1A receptors has been identified in individuals who have died by suicide (32), along with diminished quantities or affinities of somatic dendritic and postsynaptic 5-HT1A receptors (33). Recent investigations have revealed disturbances in cortical 5-HT1A receptor activity among patients with suicidal MDD (34). Importantly, significant similarities in 5-HT receptor patterns and regulation have been observed when comparing depressive symptoms with suicidal behavior, suggesting the influence of shared genetic and epigenetic pathways on both depression and suicidal conduct.

Secondly, our investigation has revealed a positive association between the risk of SA and the severity of anxiety symptoms. This finding is consistent with previous research that has identified depression as a significant risk factor for suicide and has also demonstrated the role of anxiety symptoms in elevating the risk of suicide (35, 36). Prior studies have indicated that, both before and after accounting for the level of depression, individuals with higher levels of anxiety exhibit a higher frequency of SA compared to those without anxiety symptoms (37). Furthermore, a notable 79% of patients who died by suicide either during their hospital stay or immediately after discharge displayed severe symptoms of anxiety or agitation (38). A systematic review exploring suicide as an outcome has identified comorbid anxiety disorders as a risk factor for suicide among individuals with depression (39). Additionally, several studies have observed a higher rate of SA among patients with MDD who also experience anxiety symptoms in comparison to those without anxiety symptoms (40, 41). Based on a prospective study spanning five years, it is postulated that anxiety contributes to the risk of SA through its ability to exacerbate illness severity and subsequently increase the risk of suicide (41). Serotonergic dysfunction represents a plausible biological mechanism linking anxiety symptoms to SA (42). Moreover, patients with elevated levels of anxiety face an increased likelihood of engaging in substance abuse, which further amplifies the risk of SA (43). These findings underscore the importance for clinicians to closely monitor patients with comorbid anxiety and depression, regularly assess their anxiety symptoms, and implement appropriate measures to mitigate and prevent suicidal behaviors.

Thirdly, our research findings have established a correlation between SA and TSH as well as TPOAb. Elevated levels of TSH, TgAb, and TPOAb have previously been linked to an increased risk of depression and suicide (44), indicating their potential utility as biomarkers for evaluating SA in individuals with depression. Early investigations have demonstrated that patients with MDD who have attempted suicide exhibit higher levels of TSH, which are significantly associated with SA (45). For instance, a study conducted by Berlin reported a positive correlation between SA and serum TSH levels equal to or above the upper 25th percentile of the normal range among patients with MDD (46). There is also evidence suggesting that TPOAb may contribute to an increased risk of suicide and exacerbate symptoms of anxiety and depression (47). The mechanism underlying the association between TPOAb and suicide risk can be understood by considering the following factors: TPOAb serves as a critical enzyme involved in the production of thyroid hormones and serves as an indicator of autoimmune thyroid disorders, such as Graves’ disease. It is well established that individuals with autoimmune thyroid disease have a higher prevalence of suicide. For example, a recent large-scale cohort study conducted in the Danish population revealed that even after adjusting for underlying medical and mental conditions, individuals with Graves’ disease still had a heightened risk of suicide mortality (48). Furthermore, somatostatin and serotonin, known modulators of the hypothalamic-pituitary-thyroid axis (49), establish a connection between thyroid function, depression, and suicide. While various thyroid hormones and antibodies have been associated with SA, our research suggests that TSH and TPOAb warrant particular attention as indicators of concern.

In light of the investigation into hypertension, numerous studies have delved into unraveling the relationship between high blood pressure and the peril of suicide (50, 51). A recent study revealed that a staggering 19.6% of hypertensive Chinese individuals harbored suicidal ideations (51). Our research findings demonstrate a noteworthy positive correlation between SA in patients suffering from FEDN MDD and SBP, irrespective of other influencing factors. To elucidate this connection further, Dr. Scott et al. meticulously examined data from the World Mental Health Surveys, encompassing a substantial sample size of 37,915 individuals aged 18 and above. Their study successfully established a significant and autonomous linear association between blood pressure (BP) and SA (52). Moreover, the intricate interplay between SA and hypertension was found to be subject to the influence of psychological distress (53). This entwined cycle perpetuates a reciprocal relationship wherein psychological stress and hypertension synergistically converge, potentially culminating in suicidal behavior. Notably, hypertensive patients face an escalated risk of SA, even after accounting for other physiological and psychological ailments (52). Previous investigations have postulated that dysregulation of the stress response system may be implicated in the development of hypertension and subsequent elevation in blood pressure, which has been implicated in the pathogenesis of suicide (54, 55).

Despite the satisfactory performance of our nomogram, it is important to acknowledge certain limitations. Firstly, our nomogram lacks verification in a heterogeneous population and relies solely on validation within the same training group. As a result, the obtained results may exhibit an excessive level of optimism. Secondly, this cross-sectional investigation exclusively included Han Chinese participants and was conducted solely within an outpatient clinic located in Shanxi Province, China. Therefore, additional external validation studies are crucial to ascertaining the applicability of our findings to diverse populations and other ethnic groups. Thirdly, the study exhibited an overrepresentation of female patients, which raises concerns about potential selection bias influencing the results to some extent. Fourthly, it should be noted that the current assessment outcomes may not fully meet practical expectations. The incorporation of novel biochemical markers or indicators, particularly those related to genetic factors, holds the potential to enhance the capacity of the model in future research endeavors. Fifth, the validation of the model has resulted in an AUC value of 0.821. While this indicates a relatively good performance, it falls below the threshold of 0.85, suggesting there is room for improvement. In order to enhance the model’s predictive accuracy, it is recommended to consider exploring alternative techniques, particularly those from the field of machine learning (56, 57). It is possible to refine the model and potentially achieve a higher level of accuracy. Therefore, it is recommended for future research to further explore these options and evaluate their effectiveness in improving the model’s predictive accuracy. Sixth, this study was a cross-sectional analysis, and baseline clinical characteristics, blood sample data, and the outcome of ‘suicide attempts’ were collected simultaneously. This approach is intended for diagnostic purposes to identify associated risk factors rather than to predict future occurrences. In future research, we aim to conduct longitudinal studies with follow-up periods to enhance our predictive capabilities. Finally, it is imperative to recognize the existence of potential limitations when studying patients with FEDN in this investigation. Particularly due to the overlapping presentation of depressive symptoms in patients with unipolar depression and bipolar disorder, we cannot entirely exclude the inclusion of patients whose diagnosis may transition to bipolar disorder, although we made a second diagnosis during the 3- to 6-month follow-up period and only included patients who were diagnosed with MDD at both time points.

## 5 Conclusions

We have developed and validated a novel and reliable nomogram to assess suicide risk in depressed patients, demonstrating a reasonable level of accuracy. The nomogram identifies HAMD, HAMA, TSH, Log (TPOAb), and SBP as potential risk factors for recent SA. To effectively identify high-risk patients, we provide an online version of the nomogram, accessible at https://doctorjunjunliu.shinyapps.io/dynnomapp/. However, further research is needed to externally validate the nomogram and ascertain whether timely individual interventions guided by the risk assessment tool can reduce the incidence of SA and improve treatment outcomes.

## Data availability statement

The original contributions presented in the study are included in the article/**Supplementary Material**. Further inquiries can be directed to the corresponding authors.

## Ethics statement

Ethical approval for this study was granted by the Institutional Review Board of the First Hospital, Shanxi Medical University (No. 2016-Y27). The studies were conducted in accordance with the local legislation and institutional requirements. The participants provided their written informed consent to participate in this study.

## Author contributions

JL: Formal analysis, Funding acquisition, Investigation, Methodology, Writing – original draft, Writing – review & editing. RT: Methodology, Software, Writing – original draft, Writing – review & editing. ZmL: Formal analysis, Investigation, Methodology, Writing – original draft, Writing – review & editing. YW: Conceptualization, Formal analysis, Visualization, Writing – original draft. YL: Formal analysis, Methodology, Visualization, Writing – original draft. HY: Formal analysis, Visualization, Writing – original draft. FJ: Conceptualization, Formal analysis, Methodology, Writing – original draft. XZ: Formal analysis, Methodology, Visualization, Writing – original draft. ZL: Data curation, Formal analysis, Visualization, Writing – original draft. XD: Funding acquisition, Supervision, Validation, Visualization, Writing – original draft, Writing – review & editing. ZW: Project administration, Supervision, Validation, Visualization, Writing – original draft, Writing – review & editing. XZ: Conceptualization, Investigation, Project administration, Supervision, Visualization, Writing – original draft, Writing – review & editing.

## Glossary

**Table:**

SA	suicide attempts
FEDN	first-episode drug-naïve
MDD	major depressive disorder
HAMD-17	the 17-item Hamilton Depression Scale
HAMA	the Hamilton Anxiety Scale
FBG	fasting blood glucose
ROC	receiver operating characteristic
AUC	receiver operating characteristic curve
DCA	decision curve analysis
TSH	thyroid stimulating hormone
FT3	free triiodothyronine
FT4	free thyroxine
TgAb	antithyroglobulin
TPOAb	thyroid peroxidase antibody
SBP	systolic blood pressure
DBP	diastolic blood pressure
LDL-C	low-density lipoprotein cholesterol
HDL-C	high-density lipoprotein cholesterol, TC, total cholesterol
TG	triglycerides
DSM	Diagnostic and Statistical Manual of Mental Disorders
TRIPOD	the Transparent Reporting of a Multivariable Prediction Model for Individual Prognosis or Diagnosis
BMI	body mass index
VIF	variance inflation factor
LASSO	the least absolute shrinkage and selection operator
OR	Odds Ratio
CI	Confidence Interval
