# Supplementary material for: Development and validation of a nomogram for suicide attempts in patients with first-episode drug-naïve major depressive disorder
Source: Front Psychiatry. 2024 Jun 6;15:1398733. doi: 10.3389/fpsyt.2024.1398733 (PMC11187325; doi:10.3389/fpsyt.2024.1398733)
Supplement: Supplementary file 1 [file Table_1.docx]

Supplementary Table 1. Related factors for suicide attempts according to the multivariable logistic regression model in the total patients (n = 1718).

| Variables | β | S.E | Z | *P* | OR (95%CI) |
| --- | --- | --- | --- | --- | --- |
|  |  |  |  |  |  |
| Intercept | -11.10 | 1.67 | -6.64 | **<.001** | 0.00 (0.00 ~ 0.00) |
| Age | -0.01 | 0.01 | -0.82 | 0.411 | 0.99 (0.98 ~ 1.01) |
| Duration of illness | 0.01 | 0.02 | 0.66 | 0.507 | 1.01 (0.98 ~ 1.04) |
| HAMD | 0.08 | 0.03 | 2.40 | **0.017** | 1.08 (1.01 ~ 1.15) |
| HAMA | 0.22 | 0.03 | 8.47 | **<.001** | 1.25 (1.19 ~ 1.32) |
| TSH | 0.09 | 0.04 | 2.39 | **0.017** | 1.09 (1.02 ~ 1.18) |
| Log(TPOAb) | 0.65 | 0.13 | 5.13 | **<.001** | 1.92 (1.50 ~ 2.46) |
| FT3 | -0.06 | 0.10 | -0.63 | 0.529 | 0.94 (0.78 ~ 1.14) |
| FBG | 0.04 | 0.12 | 0.36 | 0.722 | 1.04 (0.83 ~ 1.31) |
| HDL-C | -0.26 | 0.25 | -1.04 | 0.297 | 0.77 (0.47 ~ 1.26) |
| TG | -0.04 | 0.07 | -0.53 | 0.599 | 0.96 (0.83 ~ 1.11) |
| LDL-C | -0.08 | 0.09 | -0.89 | 0.372 | 0.92 (0.78 ~ 1.10) |
| BMI | -0.08 | 0.04 | -2.28 | **0.023** | 0.92 (0.86 ~ 0.99) |
| SBP | 0.03 | 0.01 | 3.54 | **<.001** | 1.03 (1.01 ~ 1.05) |
| Gender | 0.01 | 0.15 | 0.10 | 0.921 | 1.01 (0.76 ~ 1.36) |
| Education | 0.04 | 0.09 | 0.43 | 0.669 | 1.04 (0.87 ~ 1.24) |
| Marital status | -0.15 | 0.21 | -0.73 | 0.464 | 0.86 (0.57 ~ 1.29) |
| OR: Odds Ratio, CI: Confidence Interval | | | | | |
