# Supplementary material for: Development and validation of a nomogram for suicide attempts in patients with first-episode drug-naïve major depressive disorder
Source: Front Psychiatry. 2024 Jun 6;15:1398733. doi: 10.3389/fpsyt.2024.1398733 (PMC11187325; doi:10.3389/fpsyt.2024.1398733)
Supplement: Supplementary file 2 [file DataSheet_1.docx]

The statistical analysis of this study was mainly conducted using EmpowerStats statistical software（ http://www.empowerstats.com X&Y Solution, Inc., Boston, Massachusetts, USA. This software runs based on R. The following is the code and process for data analysis in this study.

## Created by EmpowerStats @ Tue, 20 Jun 23 22:42:41 +0800##

#******************** Regarding ALL Following R Code ********************

#*** COPYRIGHT (c) 2010, 2021 X&Y Solutions, ALL RIGHT RESERVED ***********

#******************* www.EmpowerStats.com *********************************

#**************************************************************************

Sys.setlocale(category = 'LC_ALL', locale = 'English_United States.1252');

.libPaths(file.path(R.home(),'library'));

library(doBy);

options(timeout=600);

library(plotrix);

library(stringi);

library(stringr);

library(survival);

library(rms);

library(nnet);

library(car);

library(mgcv);

pdfwd<-6; pdfht<-6;

load('D:/EmpowerRCH/Analysis/MDDPredictEnglish/MDDPredictEnglish.Rdata');

if (length(which(ls()=='EmpowerStatsR'))==0) EmpowerStatsR<-get(ls()[1]);

names(EmpowerStatsR)<-toupper(names(EmpowerStatsR));

originalVNAME<-names(EmpowerStatsR);

ofname<-'MDDPredictEnglish_1_tbl';

attach(EmpowerStatsR);

sink(paste(ofname,'_datastep.lst',sep=''));

print('Creating new variable: A.TPO.LOG');

A.TPO.LOG<- ifelse(A.TPO>0, log10(A.TPO), NA);

summary(A.TPO.LOG);

EmpowerStatsR<-cbind(EmpowerStatsR,A.TPO.LOG);

print('Creating new variable: HAMD.CONT');

HAMD.CONT<- HAMD;

summary(HAMD.CONT);

EmpowerStatsR<-cbind(EmpowerStatsR,HAMD.CONT);

rm(A.TPO.LOG,HAMD.CONT);

sink();

vname<-c(NA,'TRAIN','TRAIN.0','TRAIN.1','SA','SA.0','SA.1','HAMD','HAMD.24','HAMD.25','HAMD.26','HAMD.27','HAMD.28','HAMD.29','HAMD.30','HAMD.31','HAMD.32','HAMD.33','HAMD.34','HAMD.35','HAMD.36','HAMD.37','HAMD.38','HAMD.39','HAMD.40','HAMD.41','HAMA','TSH','A.TPO','SBP','A.TPO.LOG','HAMD.CONT')[-1];

vlabel<-c(NA,'TRAIN',' 0',' 1','SA',' 0',' 1','HAMD',' 24',' 25',' 26',' 27',' 28',' 29',' 30',' 31',' 32',' 33',' 34',' 35',' 36',' 37',' 38',' 39',' 40',' 41','HAMA','TSH','A.TPO','SBP','A.TPO Log','HAMD continuous')[-1];

varused4this <- c('TRAIN','SA','HAMD','HAMA','TSH','A.TPO','SBP','A.TPO.LOG','HAMD.CONT');

pkgs<-c('pROC','survivalROC','plyr','rms','rmda','Hmisc');

for (g in pkgs) {

if (!(g %in% rownames(installed.packages()))) install.packages(g,repos='https://cloud.r-project.org');

}

library(pROC);

library(survivalROC);

library(plyr);

library(rms);

library(rmda);

library(Hmisc);

WD <- EmpowerStatsR; rm(EmpowerStatsR); gc();

title<-'诊断试验与ROC分析';

WD<-subset(WD, ((!is.na(TRAIN) & (TRAIN == 1))));

wd.subset<-paste("Use subset of data: (!is.na(TRAIN) & (TRAIN == 1))");

colvname<-'SA'; colvlv<- 2;

xvname<-c('HAMD.CONT','HAMA','TSH','A.TPO.LOG','SBP');

xlv<-c(0,0,0,0,0);

cox<- 0;

timevar<- NA;

vname.start<- NA;

par3<- NA;

par1<-2;

avname<-c();

chk<- 0;

bvar<- NA;

par2<- NA;

dec<-4;

##R package## pROC survivalROC plyr rms rmda Hmisc ##R package##;

pvformat<-function(p,dec) {

pp <- sprintf(paste("%.",dec,"f",sep=""),as.numeric(p))

if (is.matrix(p)) {pp<-matrix(pp, nrow=nrow(p)); colnames(pp)<-colnames(p);rownames(pp)<-rownames(p);}

lw <- paste("<",substr("0.00000000000",1,dec+1),"1",sep="");

pp[as.numeric(p)<(1/10^dec)]<-lw

return(pp)

}

numfmt<-function(p,dec) {

if (is.list(p)) p<-as.matrix(p)

pp <- sprintf(paste("%.",dec,"f",sep=""),as.numeric(p))

if (is.matrix(p)) {pp<-matrix(pp, nrow=nrow(p));colnames(pp)<-colnames(p);rownames(pp)<-rownames(p);}

pp[as.numeric(p)>10000000]<- "inf."

pp[is.na(p) | gsub(" ","",p)==""]<- ""

pp[p=="-Inf"]<-"-Inf"

pp[p=="Inf"]<-"Inf"

return(pp)

}

rocplot<-function(x,xb) {

xlm<-c(0,1);

x1 <- 1-x$specificities; y1<-x$sensitivities;

if (x$percent) {x1<-x1/100; y1<-y1/100;}

plot(x1, y1, xlim=c(0, 1), ylim=c(0, 1), xlab="1 - Specificity", ylab="Sensitivity",

asp=0.9, mar=c(4, 4, 2, 2)+.1, mgp=c(2.5, 1, 0), col=par("col"),

lty=par("lty"), lwd=2, type="l", main=paste(xb, "\nAUC =", round(x$auc,3)));

abline(a=0,b=1,col="darkgrey",lty=1,lwd=1);

}

rocplot2<-function(x,xb,mainb) {

n=length(x); xlm<-c(0,1); ccol<-(1:n)

x1 <- 1-x[[1]]$specificities; y1<-x[[1]]$sensitivities; auc<-x[[1]]$auc

if (x[[1]]$percent) {x1<-x1/100; y1<-y1/100;}

if (n==1) {mb<-paste(xb[1],"\nAUC =", round(auc,3));

} else {mb<-mainb;}

plot(x1, y1, xlim=xlm, ylim=xlm,

xlab="1 - Specificity", ylab="Sensitivity",

asp=0.9, mar=c(4, 4, 2, 2)+.1, mgp=c(2.5, 1, 0),

col=1, lty=1, lwd=2, type="l", main=mb);

abline(a=0,b=1,col="darkgrey",lty=1,lwd=1);

for (k in (2:n)) {

auc<-c(auc,x[[k]]$auc)

par(new=T)

xk <- 1-x[[k]]$specificities; yk<-x[[k]]$sensitivities

if (x[[k]]$percent) {xk<-xk/100; yk<-yk/100;}

plot(xk, yk, xlim=xlm, ylim=xlm, xlab="", ylab="",

asp=0.9, mar=c(4, 4, 2, 2)+.1, mgp=c(2.5, 1, 0),

col=k, lty=1, lwd=2, type="l", main="");

}

if (n>1) {

mlen<-min(max(nchar(xb)),15);

mspace<-paste(rep(" ",mlen),collapse="")

xb1<-substr(paste(xb,mspace),1,mlen)

tauc<-paste(xb1,pvformat(auc,3),sep=": ")

ord<-order(auc,decreasing=TRUE)

tauc<-tauc[ord]; clr<-(1:n)[ord]

legend(0.6,0.4,tauc,title="AUC",col=clr,lty=1,lwd=1,bty="n")

}

}

diagss<-function(d,x,xb,xlb,ylb,pngf) {

cmp<- !is.na(d) & !is.na(x); d<-d[cmp]; x<-x[cmp]

d0<-d; d<-2-d; ndL <- length(levels(factor(d))); nxL <- length(levels(factor(x)));

if (ndL!=2 | nxL<2) return (c(xb,rep(" ",6)))

lr <- rep(NA,nxL)

if (nxL==2) {x<-2-x; xlb<-c(xlb[2],xlb[1])}

t0<-table(x,d); ctot<-apply(t0,2,sum); rtot<-apply(t0,1,sum)

for (i in (1:nrow(t0))) lr[i] <- (t0[i,1]/ctot[1]) / (t0[i,2]/ctot[2])

tt <- rbind(c(xb, " "," "," "),cbind(xlb,t0,format(round(lr,dec),nsmall=dec)))

if (nxL==2) {

sen <- t0[1,1]/ctot[1]; spe<- t0[2,2]/ctot[2]

ppv <- t0[1,1]/rtot[1]; npv<- t0[2,2]/rtot[2]

acc<-(t0[1,1]+t0[2,2])/(ctot[1]+ctot[2])

plr<-sen/(1-spe); nlr<-(1-sen)/spe; nnd<-1/(sen+spe-1); dor<-plr/nlr

tt<-cbind(tt,rbind(format(round(c(sen,spe,ppv,npv,acc,plr,nlr,dor,nnd),dec),nsmall=dec),matrix(" ",nrow=nxL,ncol=9)))

} else {

tmp.glm<-glm(d0~factor(x),family=binomial(link="logit"))

roc0<-roc(d0,predict(tmp.glm))

a.tss<-roc2xls(roc0,d0)

colnames(a.tss)<-c("threshold","specificity","sensitivity","accuracy","positive-LLR","negative-LLR","diagnose-OR","N-for-diagnose","postive-pv","negative-pv","a","b","c","d")

bb<-format(round(a.tss[which.max(a.tss[,2]+a.tss[,3]),c(3,2,9,10,4,5,6,7,8)],dec),nsmall=dec)

xlsfname<-paste(pngf,"_roc.xls",sep="")

write.table(a.tss,file=xlsfname,row.names=FALSE,col.names=TRUE,sep="\t",append=FALSE,quote=FALSE)

png(paste(ofname,"_raw.png",sep="")); rocplot(roc0,xb); dev.off()

pdf(paste(ofname,"_raw.pdf",sep=""), width=pdfwd, height=pdfht, family="Helvetica"); rocplot(roc0,xb); dev.off()

tt<-cbind(tt,rbind(bb,matrix(" ",nrow=(nrow(tt)-1),ncol=9)))

}

return(tt)

}

roc2xls<-function(roc0,d) {

a.tss<-t(coords(roc0,"all",ret=c("threshold", "specificity", "sensitivity"), transpose=TRUE))

bdac<-table(d); t.bd<-bdac[1]; t.ac<-bdac[2]

t.a<-a.tss[,3]*t.ac; t.c<-t.ac-t.a; t.d<-a.tss[,2]*t.bd; t.b<-t.bd-t.d;

acc<-(t.a+t.d)/(t.ac+t.bd)

plr<-(t.a/(t.a+t.c))/(t.b/(t.b+t.d))

nlr<-(t.c/(t.a+t.c))/(t.d/(t.b+t.d))

dor<-plr/nlr

nnd<-1/(t.a/(t.a+t.c)-t.b/(t.b+t.d))

ppv<-t.a/(t.a+t.b)

npv<-t.d/(t.c+t.d)

a.tss<-cbind(a.tss,acc,plr,nlr,dor,nnd,ppv,npv,t.a,t.b,t.c,t.d)

return(a.tss)

}

glm2formula<-function(mdl) {

coe<-summary(mdl)$coefficients;

tmp<-rownames(coe);

tmp<-gsub(")","=",tmp)

tmp[substr(tmp,1,7)=="factor("]<-paste(substr(tmp[substr(tmp,1,7)=="factor("],7,99),")",sep="")

tmp<-paste("*",tmp,sep="")

tmp[1]<-"";

tme<-gsub(" ","",numfmt(coe[,1],5));

tme[as.numeric(tme)>0]<-paste("+",tme[as.numeric(tme)>0],sep="")

tme<-paste(tme,tmp,sep="",collapse=" ")

if (substr(tme,1,1)=="+") tme<-substr(tme,2,999);

return(paste("logit(",colvname,") = ",tme,sep=""))

}

diagrocx1<-function(d,x,xb,pngfname) {

cmp<- !is.na(d) & !is.na(x); d<-d[cmp]; x<-x[cmp]

ndL <- length(levels(factor(d)));

tt<-c(xb,format(c(sum(d),length(d)-sum(d)),nsmall=0))

if ((min(x)==max(x)) | ndL!=2) return (c(tt,rep(" ",3)))

roc0<-roc(d,x)

a.tss<-roc2xls(roc0,d)

colnames(a.tss)<-c("threshold","specificity","sensitivity","accuracy","positive-LLR","negative-LLR","diagnose-OR","N-for-diagnose","postive-pv","negative-pv","a","b","c","d")

b.tss<-a.tss[which.max(a.tss[,2]+a.tss[,3]),]

xlsfname<-paste(pngfname,"_roc.xls",sep="")

write.table(a.tss,file=xlsfname,row.names=FALSE,col.names=TRUE,sep="\t",append=FALSE,quote=FALSE)

png(paste(pngfname,"_raw.png",sep="")); rocplot(roc0,xb); dev.off()

pdf(paste(pngfname,"_raw.pdf",sep=""), width=pdfwd, height=pdfht, family="Helvetica"); rocplot(roc0,xb); dev.off()

roc1<-try(roc(d,x,smooth=bt.smooth,ci=TRUE,boot.n=bt.times,ci.alpha=0.95))

if (length(roc1)>2) {

if (bt.smooth) {

png(paste(pngfname,".png",sep="")); rocplot(roc1,xb); dev.off()

pdf(paste(pngfname,".pdf",sep=""), width=pdfwd, height=pdfht, family="Helvetica"); rocplot(roc1,xb); dev.off()

sens.ci<-try(ci.se(roc1,specificities=seq(0,1,0.05)))

if (length(sens.ci)>1) {

png(paste(pngfname,"_ci.png",sep=""))

plot(roc1,grid=TRUE,legacy.axes=TRUE,main=paste(xb, "\nAUC = ",round(roc1$auc,3)))

plot(sens.ci,type="shape",col="lightblue")

plot(sens.ci,type="bars")

dev.off()

pdf(paste(pngfname,"_ci.pdf",sep=""), width=pdfwd, height=pdfht, family="Helvetica");

plot(roc1,grid=TRUE,legacy.axes=TRUE,main=paste(xb, "\nAUC = ",round(roc1$auc,3)))

plot(sens.ci,type="shape",col="lightblue")

plot(sens.ci,type="bars")

dev.off()

}

}

aucci<-c(numfmt(c(roc1$auc,roc1$ci[c(1,3)],b.tss[1:10]),dec),b.tss[11:14])

} else {aucci<-rep(NA,17);}

return(list(c(tt,aucci),roc0))

}

diagrocx2<-function(d,x1,x1b,x2,x2b,pngfname,k) {

cmp<- !is.na(d) & !is.na(x1) & !is.na(x2); d<-d[cmp];x1<-x1[cmp];x2<-x2[cmp]

ndL <- length(levels(factor(d)));

tt1<-c(x1b,format(c(sum(d),length(d)-sum(d)),nsmall=0))

tt2<-c(x2b,format(c(sum(d),length(d)-sum(d)),nsmall=0))

if (ndL!=2) return (rbind(c(tt1,rep(" ",3)),c(tt2,rep(" ",3))))

tt<-c("Test/model","D+","D-","AUC","AUC.low","AUC.upp");

cnm<-c("threshold", "specificity", "sensitivity","accuracy","positive-LLR","negative-LLR")

cnm<-c(cnm,"diagnose-OR","N-for-diagnose","postive-pv","negative-pv","a","b","c","d")

tt<-c(tt,cnm)

if (min(x1)!=max(x1)) {

roc0<-roc(d,x1)

a.tss1<-roc2xls(roc0,d)

colnames(a.tss1)<-cnm

b.tss<-a.tss1[which.max(a.tss1[,2]+a.tss1[,3]),]

png(paste(pngfname,"_raw1.png",sep="")); rocplot(roc0,x1b); dev.off()

pdf(paste(pngfname,"_raw1.pdf",sep=""), width=pdfwd, height=pdfht, family="Helvetica"); rocplot(roc0,x1b); dev.off()

roc1<-try(roc(d,x1,smooth=bt.smooth,ci=TRUE,boot.n=bt.times,ci.alpha=0.95))

if (length(roc1)>2) {

if (bt.smooth) {

png(paste(pngfname,"_1.png",sep="")); rocplot(roc1,x1b); dev.off()

pdf(paste(pngfname,"_1.pdf",sep=""), width=pdfwd, height=pdfht, family="Helvetica"); rocplot(roc1,x1b); dev.off()

sen1.ci<-try(ci.se(roc1,specificities=seq(0,1,0.05)))

if (length(sen1.ci)>1) {

png(paste(pngfname,"_ci1.png",sep=""))

plot(roc1,xlim=c(1.0,0.0),grid=TRUE,legacy.axes=TRUE,main=paste(x1b, "\n AUC = ",round(roc1$auc,3)))

plot(sen1.ci,type="shape",col="lightblue")

plot(sen1.ci,type="bars")

dev.off()

pdf(paste(pngfname,"_ci1.pdf",sep=""), width=pdfwd, height=pdfht, family="Helvetica");

plot(roc1,xlim=c(1.0,0.0),grid=TRUE,legacy.axes=TRUE,main=paste(x1b, "\n AUC = ",round(roc1$auc,3)))

plot(sen1.ci,type="shape",col="lightblue")

plot(sen1.ci,type="bars")

dev.off()

}

}

auc1<-c(tt1,numfmt(c(roc1$auc,roc1$ci[c(1,3)],b.tss[1:10]),dec),b.tss[11:14])

} else {auc1<-rep(NA,length(tt));}

tt1=cbind(tt,auc1)

}

if (min(x2)!=max(x2)) {

roc02<-roc(d,x2)

a.tss2<-roc2xls(roc02,d)

colnames(a.tss2)<-cnm

b.tss<-a.tss2[which.max(a.tss2[,2]+a.tss2[,3]),]

png(paste(pngfname,"_raw2.png",sep="")); rocplot(roc02,x2b); dev.off()

pdf(paste(pngfname,"_raw2.pdf",sep=""), width=pdfwd, height=pdfht, family="Helvetica"); rocplot(roc02,x2b); dev.off()

roc2<-try(roc(d,x2,smooth=bt.smooth,ci=TRUE,boot.n=bt.times,ci.alpha=0.95))

if (length(roc2)>2) {

if (bt.smooth) {

png(paste(pngfname,"_2.png",sep="")); rocplot(roc2,x2b); dev.off()

pdf(paste(pngfname,"_2.pdf",sep=""), width=pdfwd, height=pdfht, family="Helvetica");rocplot(roc2,x2b); dev.off()

sen2.ci<-try(ci.se(roc2,specificities=seq(0,1,0.05)))

if (length(sen2.ci)>1) {

png(paste(pngfname,"_ci2.png",sep=""))

plot.roc(roc2,xlim=c(1.0,0.0),grid=TRUE,legacy.axes=TRUE,main=paste(x2b, "\n AUC = ",round(roc2$auc,3)))

plot(sen2.ci,type="shape",col="lightblue")

plot(sen2.ci,type="bars")

dev.off()

pdf(paste(pngfname,"_ci2.pdf",sep=""), width=pdfwd, height=pdfht, family="Helvetica");

plot.roc(roc2,xlim=c(1.0,0.0),grid=TRUE,legacy.axes=TRUE,main=paste(x2b, "\n AUC = ",round(roc2$auc,3)))

plot(sen2.ci,type="shape",col="lightblue")

plot(sen2.ci,type="bars")

dev.off()

}

}

auc2<-c(tt2,numfmt(c(roc2$auc,roc2$ci[c(1,3)],b.tss[1:10]),dec),b.tss[11:14])

} else {auc2<-rep(NA,length(tt));}

tt1=cbind(tt1,auc2)

}

if ((min(x1)!=max(x1)) & (min(x2)!=max(x2))) {

if (nrow(a.tss1)==nrow(a.tss2)) {

a.tss<-cbind(a.tss1,a.tss2)

colnames(a.tss)<-c(paste(colnames(a.tss1),"1",sep="."), paste(colnames(a.tss2),"2",sep="."))

write.table(a.tss,file=paste(pngfname,"_roc.xls",sep=""),row.names=FALSE, col.names=TRUE, sep="\t",append=FALSE,quote=FALSE)

} else {

write.table(a.tss1,file=paste(pngfname,"_roc1.xls",sep=""),row.names=FALSE, col.names=TRUE, sep="\t",append=FALSE,quote=FALSE)

write.table(a.tss2,file=paste(pngfname,"_roc2.xls",sep=""),row.names=FALSE, col.names=TRUE, sep="\t",append=FALSE,quote=FALSE)

}

if (nbg>1) {tmp.mb<-paste(bvb,bv.lb[k],sep=": ");} else {tmp.mb<-paste("Compare 2 models");}

png(paste(pngfname,"_2raw.png",sep="")); rocplot2(list(roc0,roc02),c(x1b,x2b),tmp.mb); dev.off()

pdf(paste(pngfname,"_2raw.pdf",sep=""), width=pdfwd, height=pdfht, family="Helvetica");

rocplot2(list(roc0,roc02),c(x1b,x2b),tmp.mb); dev.off()

if (length(roc1)>2 & length(roc2)>2) {

p<-roc.test(roc1,roc2,reuse.auc=FALSE,boot.n=bt.times)$p.value

ccp<-c("P(compare)",rep(" ",2),format(round(p,dec),nsmall=dec))

ccp<-c(ccp,rep(" ",length(tt)-length(ccp)))

tt1=cbind(tt1,ccp)

if (bt.times>0) {

png(paste(pngfname,"_2smooth.png",sep="")); rocplot2(list(roc1,roc2),c(x1b,x2b),tmp.mb); dev.off()

pdf(paste(pngfname,"_2smooth.pdf",sep=""), width=pdfwd, height=pdfht, family="Helvetica");

rocplot2(list(roc1,roc2),c(x1b,x2b),tmp.mb); dev.off()

}

}

}

if (!is.matrix(tt1)) {

return(rep(" ",length(tt)));

} else {

if (ncol(tt1)>1) {return(tt1[,-1])} else {return(rep(" ",length(tt)))}

}

}

plotnomogram <- function(tmp.nom) {

tmp.nom.x<-tmp.nom[length(tmp.nom)][[1]]$x

x.mid<-(max(tmp.nom.x)+min(tmp.nom.x))/2

x.mid.dis<-abs(tmp.nom.x-x.mid)

x.setblank<-(1:length(tmp.nom.x))[-c(1,which(x.mid.dis==min(x.mid.dis)),length(tmp.nom.x))]

tmp.nom[length(tmp.nom)][[1]]$fat[x.setblank]<-""

lbl.nchar<-max(nchar(c(names(tmp.nom),"linear.predictor")))

lblfac<-0.2

if (lbl.nchar>20) lblfac<-0.3

if (lbl.nchar>30) lblfac<-0.4

plot(tmp.nom,xfrac=lblfac);

}

mdl2oddsratio<-function(mdlobj, rr="OR") {

tmps <- summary(mdlobj)$coefficients

tmpc <- cbind(tmps[,1],tmps[,1]-1.96*tmps[,2],tmps[,1]+1.96*tmps[,2])

tmpor<- cbind(exp(tmpc),tmps[,4])

colnames(tmpor) <- c(rr,"Low 95%CI","High 95%CI", "P value")

rownames(tmpor) <- rownames(tmps)

return(tmpor)

}

mat2htmltable<-function(mat, rcname=FALSE) {

if (rcname) mat<-cbind(c("",rownames(mat)),rbind(colnames(mat),mat))

t1<- apply(mat,1,function(z) paste(z,collapse="</td><td>"))

t2<- paste("<tr><td>",t1,"</td></tr>")

return(paste(t2,collapse=" "))

}

vlabelN<-(substr(vlabel,1,1)==" ");

vlabelZ<-vlabel[vlabelN];vlabelV<-vlabel[!vlabelN]

vnameV<-vname[!vlabelN];vnameZ<-vname[vlabelN]

bt.smooth<-chk; bt.times<-500;

if (is.na(par2) || par2=="") {

study.type <- "cohort";

} else {

if (par2>=1) par2 <- par2/100

if (par2>0) {study.type<-"case-control"; population.r <- par2;} else {study.type="cohort";}

}

if (length(avname)==0 & par1==3) par1==2

if (length(avname)>0) av<-WD[,avname]

if (!is.na(bvar)) {

bv<-WD[,bvar]

bvb<-vlabelV[match(bvar,vnameV)]; if (is.na(bvb)) bvb<-bvar;

bv.lv<-levels(factor(WD[,bvar]));

bv.lb<-vlabelZ[match(paste(bvar,bv.lv,sep="."),vnameZ)]

bv.lb[is.na(bv.lb)]<-bv.lv[is.na(bv.lb)]

nbg<-length(bv.lv);

} else {bv<-rep(1,nrow(WD)); bv.lv<-rep(1,nrow(WD)); nbg<-1; bvb<-"ALL";}

nx<-length(xvname); xb<-vlabelV[match(xvname,vnameV)]; xb[is.na(xb)]<-xvname[is.na(xb)];

xv<-WD[,xvname]

if (nx==1) xv<-matrix(xv,ncol=1)

colv.lv<-levels(factor(WD[,colvname]))

yb<-vlabelV[match(colvname,vnameV)]; if (is.na(yb)) yb<-colvname;

ylb<-c("0","1")

if (length(colv.lv)==2 & colv.lv[1]==0 & colv.lv[2]==1) {

yv<-WD[,colvname];

} else {

if (length(colv.lv)>=2) {

yv<-(WD[,colvname]==colv.lv[length(colv.lv)])*1; yv[is.na(WD[,colvname])]<-NA;

ylb<-c("0",paste(colvname,"=",colv.lv[length(colv.lv)],sep=""))

} else {par1==0;}

}

ofname1<-ofname;

tmp.ss<-rep(NA,13); if (nbg>1) tmp.ss<-rep(NA,14)

tmp.roc<-rep(NA,20)

dec<-4; rocc<-list(NA)

sink(paste(ofname1,".txt",sep=""))

tmp.fml<-NULL

w.00<-""

tmp1<-c("Test","1","0","ROC area(AUC)","95%CI low","95%CI upp","Best threshold","Specificity","Sensitivity")

tmp1<-c(tmp1,"Accuracy","Positive-LR","Negative-LR","Diagnose-OR","N-for-diagnose","Postive-pv","Negative-pv","a","b","c","d")

for (k in (1:nbg)) {

ncxplot<-0

if (nbg>1) {

print(paste(bvb,"=",bv.lb[k]))

w.00<-c(w.00,"</br>", paste(bvb,"=",bv.lb[k]), "</br></br>")

}

if (par1==1) {

dcas <- list()

ndca <- 0

dcaname <- NULL

for (i in (1:nx)) {

if (is.na(bvar)) {

x.tmp<-xv[,i]; y.tmp<-yv;

} else {

x.tmp<-xv[bv==bv.lv[k],i]; y.tmp<-yv[bv==bv.lv[k]]

}

cmp<-(!is.na(x.tmp) & !is.na(y.tmp))

x.tmp<-x.tmp[cmp]; y.tmp<-y.tmp[cmp]; WDTMP <- as.data.frame(cbind(y.tmp,x.tmp))

singleChk.x<-(max(x.tmp)!=min(x.tmp))

if (length(levels(factor(y.tmp)))==2 & singleChk.x) {

pngf<-paste(ofname1,xvname[i],sep="_")

if (nbg>1) pngf<-paste(pngf,bv.lv[k],sep="_")

if (xlv[i]==0) {

tmp.xb<-xb[i]; if (nbg>1) tmp.xb<-paste(tmp.xb," (",bvb,"=",bv.lb[k],")",sep="")

tmp<-diagrocx1(y.tmp,x.tmp,tmp.xb,pngf)

tmp.roc<-rbind(tmp.roc,tmp[[1]])

ncxplot<-ncxplot+1

rocc[[ncxplot]]<-tmp[[2]]

if (ncxplot==1) {xxb<-tmp.xb;} else {xxb<-c(xxb,tmp.xb);}

ndca <- ndca + 1

dcas[[ndca]] <- decision_curve(y.tmp~x.tmp, data=WDTMP, study.design="cohort", policy="opt-in", bootstraps = 50)

write.table(dcas[[ndca]]$derived.data,file=paste(pngf,xvname[i],"dca.xls",sep="_"),col.names=TRUE,row.names=FALSE,sep="\t")

if (is.null(dcaname)) {dcaname <- xvname[i];} else {dcaname<-c(dcaname, xvname[i]);}

} else {

xi.lv<-levels(factor(x.tmp))

tmp.xlb<-vlabelZ[match(paste(xvname[i],xi.lv,sep="."),vnameZ)]

tmp.xlb[is.na(tmp.xlb)]<-xi.lv[is.na(tmp.xlb)]

tmp<-diagss(y.tmp,x.tmp,xb[i],tmp.xlb,ylb,pngf)

if (nbg>1) tmp<-cbind(bv.lb[k],tmp)

tmp.ss<-rbind(tmp.ss,tmp)

}

}

rm(WDTMP)

}

if (ncxplot>1) {

pngf<-ofname1; if (nbg>1) pngf<-paste(pngf,bv.lv[k],sep="_")

if (nbg>1) {tmp.mb<-paste(bvb,bv.lb[k],sep=": ");} else {tmp.mb<-paste("ROC curve for", yb);}

png(paste(pngf,"_rocs.png",sep=""),width=640,height=640); rocplot2(rocc,xxb,tmp.mb); dev.off()

pdf(paste(pngf,"_rocs.pdf",sep=""),width=pdfwd, height=pdfht, family="Helvetica");rocplot2(rocc,xxb,tmp.mb); dev.off()

}

if (is.matrix(tmp.ss)) {

tmp0<-c("Test","1","0","Likelihood ratio(LR)","Sensitivity","Specificity","Positive pv","Negative pv","Accuracy","Positive-LR","Negative-LR","Diagnosis OR","Number for diagnose")

if (nbg>1) tmp0<-c(bvb,tmp0)

tmp.ss<-rbind(tmp0,tmp.ss[-1,])

w.00<-c(w.00,"</br>Sensitivity and Specificity for diagnositic test</br><table border=3>",mat2htmltable(tmp.ss),"</table>")

}

if (is.matrix(tmp.roc)) {

tmp.roc<-rbind(tmp1,tmp.roc[-1,])

tmp.roc1<-tmp.roc[,(1:9)]

tmp.roc2<-tmp.roc[,-(2:6)]

w.00<-c(w.00,"</br>ROC analysis for continuous predictor</br><table border=3>",mat2htmltable(tmp.roc1),"</table>")

w.00<-c(w.00,"</br>Best threshold analysis</br><table border=3>",mat2htmltable(tmp.roc2),"</table>")

}

if (ndca>0) {

png(paste(pngf,"_dca.png",sep=""),width=960,height=840);

if (study.type=="cohort") {

plot_decision_curve(dcas, curve.names = dcaname, col = (2:(ndca+1)), confidence.intervals = FALSE)

} else {

plot_decision_curve(dcas, curve.names = dcaname, col = (2:(ndca+1)), confidence.intervals = FALSE,

study.design = "case-control", population.prevalence = population.r)

}

dev.off()

pdf(paste(pngf,"_dca.pdf",sep=""),width=pdfwd*1.5, height=pdfht*1.5, family="Helvetica");

if (study.type=="cohort") {

plot_decision_curve(dcas, curve.names = dcaname, col = (2:(ndca+1)), confidence.intervals = FALSE)

} else {

plot_decision_curve(dcas, curve.names = dcaname, col = (2:(ndca+1)), confidence.intervals = FALSE,

study.design = "case-control", population.prevalence = population.r)

}

dev.off()

}

}

if (par1==2) {

WD0<-cbind(yv,xv); colnames(WD0)<-c(colvname,xvname);

if (nbg>1) WD0<-WD0[bv==bv.lv[k],]

cmp<-(apply(is.na(WD0),1,sum)==0)

WD0<-as.data.frame(WD0[cmp,])

if (length(levels(factor(WD0[,1])))==2) {

xv1.1<-xvname

xv1.1[xlv>2]<-paste("factor(",xv1.1[xlv>2],")",sep="")

singleChk<- apply(cbind(WD0[,xvname],1),2,function(x) return(max(x,na.rm=TRUE)-min(x,na.rm=TRUE)!=0))

fml.1<-paste(colvname,"~",paste(xv1.1[singleChk],collapse="+"))

tmp.glm<-try(glm(formula(fml.1),family=binomial(link="logit"),data=WD0))

pngf<-paste(ofname1,"model",sep="_")

if (nbg>1) pngf<-paste(pngf,bv.lv[k],sep="_")

if (substr(tmp.glm[[1]][1],1,5)!="Error") {

print(summary(tmp.glm)); print(mdl2oddsratio(tmp.glm))

tmp.lrm<-try(lrm(formula(fml.1),data=WD0))

if (substr(tmp.lrm[[1]][1],1,5)!="Error") {

tmp.dst<-datadist(WD0)

options(datadist='tmp.dst')

tmp.nom<-nomogram(tmp.lrm, fun=function(x)1/(1+exp(-x)),funlabel=yb)

print("Nomogram"); print(tmp.nom)

png(paste(pngf,"_nom.png",sep=""),width=960,height=840); plotnomogram(tmp.nom); dev.off()

pdf(paste(pngf,"_nom.pdf",sep=""),width=pdfwd*1.5, height=pdfht*1.5, family="Helvetica");plotnomogram(tmp.nom); dev.off()

}

x.tmp<-predict(tmp.glm)

y.tmp<-WD0[,1]

tmp.xb<-"Model";

tmpi<-glm2formula(tmp.glm)

if (nbg>1) {

tmp.xb<-paste(tmp.xb," (",bvb,"=",bv.lb[k],")",sep="")

tmpi<-paste("</br>",bvb,"=",bv.lb[k],":</br>",tmpi)

}

tmp.fml<-c(tmp.fml,tmpi)

tmp<-diagrocx1(y.tmp,x.tmp,tmp.xb,pngf)

tmp.roc<-cbind(tmp.roc,tmp[[1]])

dca0 <- decision_curve(formula(fml.1), data=WD0, study.design="cohort", policy="opt-in", bootstraps = 50)

png(paste(pngf,"_dca.png",sep=""),width=960,height=840);

if (study.type=="cohort") {

plot_decision_curve(dca0, curve.names="Model", confidence.intervals = FALSE, col="red")

} else {

plot_decision_curve(dca0, curve.names="Model", confidence.intervals = FALSE, col="red",

study.design = "case-control", population.prevalence = population.r)

}

dev.off()

pdf(paste(pngf,"_dca.pdf",sep=""),width=pdfwd*1.5, height=pdfht*1.5, family="Helvetica");

if (study.type=="cohort") {

plot_decision_curve(dca0, curve.names="Model", confidence.intervals = FALSE, col="red")

} else {

plot_decision_curve(dca0, curve.names="Model", confidence.intervals = FALSE, col="red",

study.design = "case-control", population.prevalence = population.r)

}

dev.off()

write.table(dca0$derived.data,file=paste(pngf,"_dca.xls",sep=""),col.names=TRUE,row.names=FALSE,sep="\t")

} else {tmp.roc<-"Model error!";}

rm(WD0)

}

if (is.matrix(tmp.roc)) {

tmp.roc<-cbind(tmp1,tmp.roc[,-1])

} else {w.00<-c(w.00,"</br>",tmp.roc);}

}

if (par1==3) {

WD0<-cbind(yv,xv,av); colnames(WD0)<-c(colvname,xvname,avname)

if (nbg>1) WD0<-WD0[bv==bv.lv[k],]

cmp<-(apply(is.na(WD0),1,sum)==0)

WD0<-as.data.frame(WD0[cmp,])

if (length(levels(factor(WD0[,1])))==2) {

xv1<-xvname

xv1[xlv!=0]<-paste("factor(",xv1[xlv!=0],")",sep="")

singleChk.xv1 <- apply(cbind(WD0[,xvname],1),2,function(x) return(max(x)-min(x)!=0))

xv1<- xv1[singleChk.xv1];

fml<-paste(colvname,"~",paste(xv1,collapse="+"))

tmp.glm<-try(glm(formula(fml),family=binomial(link="logit"),data=WD0))

if (substr(tmp.glm[[1]][1],1,5)!="Error") {

or.tb1<- numfmt(mdl2oddsratio(tmp.glm),4)

print(summary(tmp.glm)); print(or.tb1)

x.tmp<-predict(tmp.glm)

y.tmp<-WD0[,1]

av1<-avname

av1[alv!=0]<-paste("factor(",av1[alv!=0],")",sep="")

singleChk.av1 <- apply(cbind(WD0[,avname],1),2,function(x) return(max(x)-min(x)!=0))

av1<- av1[singleChk.av1];

fml2<-paste(colvname,"~",paste(av1,collapse="+"))

tmp.glm2<-try(glm(formula(fml2),family=binomial(link="logit"),data=WD0))

if (substr(tmp.glm2[[1]][1],1,5)!="Error") {

or.tb2<- numfmt(mdl2oddsratio(tmp.glm2),4)

print(summary(tmp.glm2)); print(or.tb2)

x.tmp2<-predict(tmp.glm2)

pngf<-paste(ofname1,"model2",sep="_")

if (nbg>1) pngf<-paste(pngf,bv.lv[k],sep="_")

tmp.x1b<-"Model1"; if (nbg>1) tmp.x1b<-paste(tmp.x1b,"(",bvb,"=",bv.lb[k],")",sep="")

tmp.x2b<-"Model2"; if (nbg>1) tmp.x2b<-paste(tmp.x2b,"(",bvb,"=",bv.lb[k],")",sep="")

tmp.fml<-glm2formula(tmp.glm)

tmp.fml2<-glm2formula(tmp.glm2)

tmp.roc<-cbind(tmp.roc,diagrocx2(y.tmp,x.tmp,tmp.x1b,x.tmp2,tmp.x2b,pngf,k))

impr<-improveProb(x.tmp>=as.numeric(tmp.roc[7,2]), x.tmp2>=as.numeric(tmp.roc[7,3]), y.tmp)

print(table(x.tmp>=as.numeric(tmp.roc[7,2]), x.tmp2>=as.numeric(tmp.roc[7,3]), y.tmp));

print(impr);

w.nrp<-c("Increase for events (1)", "Increase for non-events (2)", "Decrease for events (3)", "Decrease for non-events (4)")

w.nrp<-cbind(w.nrp, numfmt(unlist(impr[c("pup.ev","pup.ne","pdown.ev","pdown.ne")]), dec))

nri<-matrix(unlist(impr[c("nri", "nri.ev", "nri.ne", "idi", "se.nri", "se.nri.ev", "se.nri.ne", "se.idi")]),ncol=2)

nri<-cbind(nri, nri[,1]/nri[,2])

nri<-cbind(nri, 2-2*pnorm(abs(nri[,3])))

nri<-cbind(nri, nri[,1]-1.96*nri[,2], nri[,1]+1.96*nri[,2])

w.nri<-numfmt(nri,dec)

w.nri[,4]<-pvformat(nri[,4],4)

w.nri<-rbind(c("Estimate","SE","Z","P value","95% Lower","95% Upper"), w.nri)

w.nri<-cbind(c("","NRI (1-3+4-2)","NRI for events (1-3)","NRI for non-events (4-2)","IDI"),w.nri)

dca1 <- decision_curve(formula(fml), data=WD0, study.design="cohort", policy="opt-in", bootstraps = 50)

dca2 <- decision_curve(formula(fml2), data=WD0, study.design="cohort", policy="opt-in", bootstraps = 50)

png(paste(pngf,"_dca.png",sep=""),width=960,height=840);

if (study.type=="cohort") {

plot_decision_curve(list(dca1, dca2), curve.names = c("Model 1", "Model 2"), col = c("blue", "red"), confidence.intervals = FALSE)

} else {

plot_decision_curve(list(dca1, dca2), curve.names = c("Model 1", "Model 2"), col = c("blue", "red"), confidence.intervals = FALSE,

study.design = "case-control", population.prevalence = population.r)

}

dev.off()

pdf(paste(pngf,"_dca.pdf",sep=""),width=pdfwd*1.5, height=pdfht*1.5, family="Helvetica");

if (study.type=="cohort") {

plot_decision_curve(list(dca1, dca2), curve.names = c("Model 1", "Model 2"), col = c("blue", "red"), confidence.intervals = FALSE)

} else {

plot_decision_curve(list(dca1, dca2), curve.names = c("Model 1", "Model 2"), col = c("blue", "red"), confidence.intervals = FALSE,

study.design = "case-control", population.prevalence = population.r)

}

dev.off()

write.table(dca1$derived.data,file=paste(pngf,"_dca1.xls",sep=""),col.names=TRUE,row.names=FALSE,sep="\t")

write.table(dca2$derived.data,file=paste(pngf,"_dca2.xls",sep=""),col.names=TRUE,row.names=FALSE,sep="\t")

} else {tmp.roc<-"Model 2 error!";}

} else {tmp.roc<-"Model 1 error!";}

rm(WD0)

}

if (is.matrix(tmp.roc)) {

tmp.roc<-cbind(tmp1,tmp.roc[,-1])

w.00<-c(w.00,"</br>Predictive model:</br>","Model 1：",tmp.fml,"</br>","Model 2: ",tmp.fml2)

w.00<-c(w.00,"</br></br>Compare 2 predictive models (ROC Curve)</br><table border=3>",mat2htmltable(tmp.roc),"</table>")

w.00<-c(w.00,"</br></br>Based on best threshold in the above table:</br>Net Reclassification Index, Integrated Discrimination Index")

w.00<-c(w.00,"</br><table border=3>",mat2htmltable(w.nrp),"</table>")

w.00<-c(w.00,"</br><table border=3>",mat2htmltable(w.nri),"</table>")

w.00<-c(w.00,"</br>Model 1</br><table border=3>",mat2htmltable(or.tb1,TRUE),"</table>")

w.00<-c(w.00,"</br>Model 2</br><table border=3>",mat2htmltable(or.tb2,TRUE),"</table>")

} else {w.00<-c(w.00,"</br>",tmp.roc);}

}

}

sink()

w<-c("<!DOCTYPE html><html lang='zh'><head><meta charset='utf-8'></head><body>")

w<-c(w,"<h2>",title,"</h2>")

w<-c(w,"</br>结局变量:", yb, "</br>")

if (par1==2) {

w <-c(w,"</br>Predictive model:</br>",tmp.fml)

tmp1<-c("检测项目","1","0","ROC面积(AUC)","95% 区间下限","95% 区间上限","最佳阈值","特异度","敏感度")

tmp1<-c(tmp1,"准确度","阳性似然比","阴性似然比","诊断比值比","诊断需要检测数","阳性预测值","阴性预测值","a","b","c","d");

tmp.roc<-cbind(tmp1,tmp.roc[,-1])

w<-c(w,"</br></br>Model ROC curve and best threshold analysis</br><table border=3>",mat2htmltable(tmp.roc),"</table>")

} else {

w<-c(w,w.00)

w<-c(w,"</br>Sensitivity: 敏感度; Specificity: 特异度; Positive pv: 阳性预测值; Negative pv: 阴性预测值; Accuracy: 准确度; Positive-LR: 阳性似然比; Negative-LR: 阴性似然比; Diagnosis OR: 诊断比值比; Number for diagnose: 诊断需要检测数 </br>")

}

if (bt.smooth) w<-c(w,paste("</br>AUC 可信区间与显著性检验采用非参数的重复采样方法(Bootstrap resampling times=",bt.times,")"))

w<-c(w,"</br></br>最佳阈值取敏感度+特异度最大的分界值。各分界点对应的敏感度特异保存在ROC输出文件（.xls）里")

w<-c(w,wd.subset)

w<-c(w,paste("</br></br>Created by EmpowerStats (www.empowerstats.com) and R on",Sys.Date()))

w<-c(w,"</body></html>")

fileConn<-file(paste(ofname1,".htm",sep="")); writeLines(w, fileConn)

## Created by EmpowerStats @ Tue, 20 Jun 23 22:46:15 +0800##

#******************** Regarding ALL Following R Code ********************

#*** COPYRIGHT (c) 2010, 2021 X&Y Solutions, ALL RIGHT RESERVED ***********

#******************* www.EmpowerStats.com *********************************

#**************************************************************************

Sys.setlocale(category = 'LC_ALL', locale = 'English_United States.1252');

.libPaths(file.path(R.home(),'library'));

library(doBy);

options(timeout=600);

library(plotrix);

library(stringi);

library(stringr);

library(survival);

library(rms);

library(nnet);

library(car);

library(mgcv);

pdfwd<-6; pdfht<-6;

load('D:/EmpowerRCH/Analysis/MDDPredictEnglish/MDDPredictEnglish.Rdata');

if (length(which(ls()=='EmpowerStatsR'))==0) EmpowerStatsR<-get(ls()[1]);

names(EmpowerStatsR)<-toupper(names(EmpowerStatsR));

originalVNAME<-names(EmpowerStatsR);

ofname<-'MDDPredictEnglish_2_tbl';

attach(EmpowerStatsR);

sink(paste(ofname,'_datastep.lst',sep=''));

print('Creating new variable: A.TPO.LOG');

A.TPO.LOG<- ifelse(A.TPO>0, log10(A.TPO), NA);

summary(A.TPO.LOG);

EmpowerStatsR<-cbind(EmpowerStatsR,A.TPO.LOG);

print('Creating new variable: HAMD.CONT');

HAMD.CONT<- HAMD;

summary(HAMD.CONT);

EmpowerStatsR<-cbind(EmpowerStatsR,HAMD.CONT);

rm(A.TPO.LOG,HAMD.CONT);

sink();

vname<-c(NA,'TRAIN','TRAIN.0','TRAIN.1','SA','SA.0','SA.1','HAMD','HAMD.24','HAMD.25','HAMD.26','HAMD.27','HAMD.28','HAMD.29','HAMD.30','HAMD.31','HAMD.32','HAMD.33','HAMD.34','HAMD.35','HAMD.36','HAMD.37','HAMD.38','HAMD.39','HAMD.40','HAMD.41','HAMA','TSH','A.TPO','SBP','A.TPO.LOG','HAMD.CONT')[-1];

vlabel<-c(NA,'TRAIN',' 0',' 1','SA',' 0',' 1','HAMD',' 24',' 25',' 26',' 27',' 28',' 29',' 30',' 31',' 32',' 33',' 34',' 35',' 36',' 37',' 38',' 39',' 40',' 41','HAMA','TSH','A.TPO','SBP','A.TPO Log','HAMD continuous')[-1];

varused4this <- c('TRAIN','SA','HAMD','HAMA','TSH','A.TPO','SBP','A.TPO.LOG','HAMD.CONT');

pkgs<-c('glmnet','glmnetUtils','mfp','missForest','dcurves','pROC','ROCR','Amelia','RANN','skimr','caret','gbm');

for (g in pkgs) {

if (!(g %in% rownames(installed.packages()))) install.packages(g,repos='https://cloud.r-project.org');

}

library(glmnet);

library(glmnetUtils);

library(mfp);

library(missForest);

library(dcurves);

library(pROC);

library(ROCR);

library(Amelia);

library(RANN);

library(skimr);

library(caret);

library(gbm);

WD <- EmpowerStatsR; rm(EmpowerStatsR); gc();

title<-'预测模型构建与验证';

wd.subset='';

colvname<-'SA'; colvlv<- 2;

xvname<-c('HAMD.CONT','HAMA','TSH','A.TPO.LOG','SBP');

sxf<-c(0,0,0,0,0);

xlv<-c(0,0,0,0,0);

timevar<- NA;

par1<-'TRAIN==1';

cbl<-c(0,2,3,5);

par2<-'automatic set';

bvar<- NA;

dec<-4;

##R package## glmnet glmnetUtils mfp missForest dcurves pROC ROCR Amelia RANN skimr caret gbm ##R package##;

pvformat<-function(p,dec) {

pp <- sprintf(paste("%.",dec,"f",sep=""),as.numeric(p))

if (is.matrix(p)) {pp<-matrix(pp, nrow=nrow(p)); colnames(pp)<-colnames(p);rownames(pp)<-rownames(p);}

lw <- paste("<",substr("0.00000000000",1,dec+1),"1",sep="");

pp[as.numeric(p)<(1/10^dec)]<-lw

return(pp)

}

numfmt<-function(p,dec) {

if (is.list(p)) p<-as.matrix(p)

pp <- sprintf(paste("%.",dec,"f",sep=""),as.numeric(p))

if (is.matrix(p)) {pp<-matrix(pp, nrow=nrow(p));colnames(pp)<-colnames(p);rownames(pp)<-rownames(p);}

pp[as.numeric(p)>10000000]<- "inf."

pp[is.na(p) | gsub(" ","",p)==""]<- ""

pp[p=="-Inf"]<-"-Inf"

pp[p=="Inf"]<-"Inf"

return(pp)

}

mat2htmltable<-function(mat, rc.name=0, dec.fmt=NA) {

if (!is.na(dec.fmt) & rc.name==1) mat<-numfmt(mat,dec.fmt)

if (rc.name==1) mat<-rbind(c(" ",colnames(mat)),cbind(rownames(mat),mat))

t1<- apply(mat,1,function(z) paste(z,collapse="</td><td>"))

t2<- paste(paste("<tr><td>",t1,"</td></tr>"),collapse="")

return(paste("<table border=3>", t2, "</table>"))

}

trim <- function (x) gsub("^\\s+|\\s+$", "", x)

vifSelect<-function(dfr, t=5, fix=4){

if(!(class(dfr)[1]=="data.frame")) dfr<-data.frame(dfr)

vnames<-names(dfr); nvar<-length(vnames);

if (!is.na(fix)) {v4slt<-(1:nvar)[-fix];} else {v4slt<-(1:nvar);}

vifmat<-matrix(NA,nrow=nvar,ncol=1)

vifmax<-100; v.rm<-0; k<-1

while (vifmax>=t) {

vnum <-(0:nvar)[-match(v.rm,(0:nvar))]

for (v in vnum) {

fml <- paste(vnames[v],"~",paste(vnames[vnum[vnum!=v]],collapse="+"))

lm.mdl<-summary(lm(formula(fml),data=dfr));

print(fml);print(lm.mdl)

vifmat[v,k]<-round(1/(1-lm.mdl$r.squared),1)

}

vifmax<-max(as.numeric(vifmat[v4slt,k]),na.rm=TRUE)

if (vifmax<t) break

v.rm<-c(v.rm,v4slt[which(as.numeric(vifmat[v4slt,k])==vifmax)[1]])

k<-k+1; vifmat<-cbind(vifmat,NA);

}

slt.vv<-!is.na(vifmat[v4slt,k])

vif.Rm<-vnames[is.na(vifmat[,k])]

vifmat <- rbind(c(" ",paste("Step",(1:k))),cbind(vnames,vifmat))

return(list(slt.vv,vif.Rm,vifmat))

}

factor2dummy<-function(v, v.name, keepNA=FALSE, allv=TRUE) {

vl<-names(sort(table(v,useNA="ifany"))); if (!allv) vl<-vl[-1]

nc<-length(vl);nr<-length(v)

new.name<-paste(v.name,gsub("[-+ ]","_",vl),sep=".")

d.out<-matrix(0,nrow=nr,ncol=nc)

for (i in 1:nc) {

if (is.na(vl[i])) {d.out[is.na(v),i]<-1;} else {d.out[v==vl[i],i]<-1;}

}

if (keepNA) {

d.out[is.na(v),]<-NA;

if (anyNA(vl)) {d.out<-d.out[,!is.na(vl)]; new.name<-new.name[!is.na(vl)];

} else if (!allv) {d.out<-d.out[,-1]; new.name<-new.name[-1];}

}

if (!is.matrix(d.out)) d.out<-matrix(d.out,ncol=1)

colnames(d.out)<-new.name; return(d.out);

}

createXmatrix<-function(xxv,xxl,WD,allv=TRUE) {

nx<-length(xxv); xx.mat<-NA;

for (i in 1:nx) {

v.name<-xxv[i]; v<-WD[,v.name];

if (xxl[i]>0) {

xx.mat<-cbind(xx.mat,factor2dummy(v,v.name,FALSE,allv));

} else {

v.tmp<-cbind(v,rep(0,length(v)));

v.tmp[is.na(v),1]<-median(v,na.rm=TRUE); v.tmp[is.na(v),2]<-1;

colnames(v.tmp)<-c(v.name,paste(v.name,"NA",sep="."))

xx.mat<-cbind(xx.mat,v.tmp)

if (sum(is.na(v))==0) xx.mat<-xx.mat[,-ncol(xx.mat)]

}

}

return(xx.mat[,-1])

}

mrocplot<-function(roclist,roclb) {

Encoding(roclb)<-"UTF-8";

n=length(roclist); xlm<-c(0,1); ccol<-(1:n)

x<-roclist[[1]][[1]]; y<-roclist[[1]][[2]]; auc<-roclist[[1]][[3]]

plot(x, y, xlim=xlm, ylim=xlm,

xlab="False positive rate", ylab="True positive rate", asp=0.9, mar=c(4, 4, 2, 2)+.1, mgp=c(2.5, 1, 0),

col=1, lty=1, lwd=2, type="l", main="ROC Curves");

abline(a=0,b=1,col="darkgrey",lty=1,lwd=1);

if (n>1) {

for (k in (2:n)) {

x<-roclist[[k]][[1]]; y<-roclist[[k]][[2]]; auc<-c(auc, roclist[[k]][[3]])

par(new=T)

plot(x, y, xlim=xlm, ylim=xlm, xlab="", ylab="",asp=0.9, mar=c(4, 4, 2, 2)+.1, mgp=c(2.5, 1, 0),

col=k, lty=1, lwd=2, type="l", main="");

}

mlen<-min(max(nchar(roclb)),15);

mspace<-paste(rep(" ",mlen),collapse="")

xb1<-substr(paste(roclb,mspace),1,mlen)

tauc<-paste(xb1,pvformat(auc,3),sep=": ")

ord<-order(auc,decreasing=TRUE)

tauc<-tauc[ord]; clr<-(1:n)[ord]

legend(0.6,0.4,tauc,title="AUC",col=clr,lty=1,lwd=1,bty="n")

} else {

legend(0.6,0.4,paste("AUC:",pvformat(auc,3)),title="",bty="n")

}

}

coe2formula<-function(coe) {

tmp<-strsplit(rownames(coe),"\\*");

if (sum(is.na(coe[,1]))>0) coe<-coe[!is.na(coe[,1]),]

if (!is.matrix(coe)) return ("");

tmp<-unlist(lapply(tmp, function(x) {

for (i in 1:length(x)) {

x[i]<-trimws(x[i])

mfpi<-match(x[i], mfp.newname)

if (!is.na(mfpi)) {x[i]<-mfp.formula[mfpi];

} else {

catgi<-match(x[i], xx[xx.xlv>0])

if (!is.na(catgi)) {

tmpvname<-xx.xvname[xx.xlv>0][catgi]

x[i]<- paste("(",str_replace(x[i], paste(tmpvname,".",sep=""), paste(tmpvname,"=",sep="")),")",sep="")

}

}

}

return (paste(x,collapse="*"))

}))

tmp<-paste("*",tmp,sep="")

if (grepl(tmp[1],"Intercept")) tmp[1]<-"";

tme<-trimws(numfmt(coe[,1],5));

tme[as.numeric(tme)>0]<-paste("+",tme[as.numeric(tme)>0],sep="")

tme<-paste(tme,tmp,sep="",collapse=" ")

if (substr(tme,1,1)=="+") tme<-substr(tme,2,999);

return(tme)

}

calibratePlot<-function(y,p,pngfname,mainb="") {

y<-y[!is.na(y) && !is.na(p)]; p<-p[!is.na(y) && !is.na(p)];

if (length(levels(factor(p))) < 20) return (0);

gam1 <- try(glm(y ~ splines::ns(p,df=4),family=binomial))

if (substr(gam1[[1]][1],1,5)!="Error") {

x <- seq(min(p), max(p), length = 200)

yy <- predict(gam1, newdata = data.frame(p = x), se.fit=TRUE, type="response")

se.lower <- yy$fit - 2 * yy$se.fit; se.lower[se.lower < 0] <- 0

se.upper <- yy$fit + 2 * yy$se.fit; se.upper[se.upper > 1] <- 1

xlim <- range(se.lower, se.upper, x)

ylim <- range(se.lower, se.upper, x)

png(paste(pngfname,".png",sep=""));

plot(0,0,type="n",xlab="Predicted probability",ylab="Observed probability",xlim=xlim,ylim=ylim,main=mainb)

polygon(c(x, rev(x), x[1L]), c(se.lower, rev(se.upper), se.lower[1L]),col="lightyellow",border=NA,density=NULL)

lines(x, yy$fit, col="black"); quantile.rug(p, side=1); abline(0, 1, col="red")

dev.off()

pdf(paste(pngfname,".pdf",sep=""),width=pdfwd, height=pdfht, family="GB1")

plot(0,0,type="n",xlab="Predicted probability",ylab="Observed probability",xlim=xlim,ylim=ylim,main=mainb)

polygon(c(x, rev(x), x[1L]), c(se.lower, rev(se.upper), se.lower[1L]),col="lightyellow",border=NA,density=NULL)

lines(x, yy$fit, col="black"); quantile.rug(p, side=1); abline(0, 1, col="red")

dev.off()

}

}

options(warn=-1)

vif.CUT <- 10;

unique.CUT <- 10;

freq.CUT <- 19;

set.seed(12345)

if (length(xvname)<1 | is.na(colvname)) stop("No predictors or outcome")

vlabelN<-(substr(vlabel,1,1)==" ");

vlabelZ<-vlabel[vlabelN];vlabelV<-vlabel[!vlabelN]

vnameV<-vname[!vlabelN];vnameZ<-vname[vlabelN]

allvname<-c(colvname,xvname,timevar); allvname<-allvname[!is.na(allvname)]

WD[,colvname]<-factor(WD[,colvname])

for (v in xvname[xlv>0]) WD[,v]<-factor(WD[,v])

xxv<-xvname; xxl<-xlv; xxf<-sxf;

w0<-"";

xb<-vlabelV[match(xvname,vnameV)]; xb[is.na(xb)]<-xvname[is.na(xb)];

yb<-vlabelV[match(colvname,vnameV)]; if (is.na(yb)) yb<-colvname;

## remove non-numeric variables

isCH<-apply(WD[,xxv],2,function(x) return(sum(is.na(as.numeric(x)))>sum(is.na(x))))

if (sum(isCH)>0) {

w0<-c(w0,"<p>Following non-numeric predictors were removed:", paste(xxv[isCH],sep=" "),"</p>")

xxv<-xxv[!isCH]; xxl<-xxl[!isCH]; xxf<-xxf[!isCH];

}

## split train and test data

if (is.na(par1) || par1=="") par1<-100

if (is.numeric(par1)) {

if (par1 < 100) {

idx.TRAIN <- createDataPartition(WD[,colvname], times=1, p=par1/100, list = FALSE)

WDT <- WD[idx.TRAIN, allvname];

WDV <- WD[-idx.TRAIN,allvname];

} else {

WDT <- WD[,allvname]

WDV <- NULL

}

} else {

t<-strsplit(par1,"=")[[1]]

splitVNAME<-trimws(toupper(t[1]))

splitVAL<-trimws(t[length(t)])

yn.TRAIN<-WD[,splitVNAME]==splitVAL

WDT<-WD[yn.TRAIN, allvname]

WDV<-WD[!yn.TRAIN,allvname]

}

WDD<-rbind(WDT,WDV); rowTrain<-c(rep(TRUE,nrow(WDT))); rm(WDT);

if (!is.null(WDV)) {rowTrain<-c(rowTrain,rep(FALSE,nrow(WDV))); rm(WDV);}

png(paste(ofname,"_missing.png",sep="")); missmap(WDD); dev.off()

WDY<-factor(WDD[,1]); WDD<-WDD[,-1]

vfac.COL<- match(xxv[xxl>0],colnames(WDD))

if (length(vfac.COL)<ncol(WDD)) {

X.mean<-apply(cbind(1,WDD[,-vfac.COL]),2,function(x) mean(x,na.rm=TRUE))

X.std <- apply(cbind(1,WDD[,-vfac.COL]),2,function(x) sd(x,na.rm=TRUE))

X.oo<-cbind(c("","Mean","SD"),rbind(c("",xxv[xxl==0]),numfmt(X.mean,4),numfmt(X.std,4)))[,-2]

w0<-c(w0,"<p>Mean and SD for continuous predictors:",mat2htmltable(X.oo),"</p>")

}

##missForest

if (sum(cbl==0)>0 && anyNA(WDD[,xxv])) {

mistmp<-try(missForest(WDD[,xxv], maxiter=10, ntree=50, parallelize='no'));

if (substr(mistmp[1],1,5) != "Error") WDD<-mistmp$ximp

}

##dummy Vars

WDD<-createXmatrix(xxv,xxl,WDD)

xx<-colnames(WDD)

## near Zero Variance check on training data

nzv<- nearZeroVar(WDD[rowTrain,],freqCut=freq.CUT,uniqueCut=unique.CUT,saveMetrics=TRUE)

nzv[,c(1,2)]<-numfmt(nzv[,c(1,2)],2)

w0<-c(w0,"<p>Near zero variance check on training data:",mat2htmltable(nzv,1),"</p>")

nzvXV<-rownames(nzv[nzv[,'nzv'],])

if (length(nzvXV)>0) {

w0<-c(w0,"<p>Following variables were removed from predictors:", paste(nzvXV,collapse=" "),"</p>")

xx<-rownames(nzv[!nzv[,'nzv'],]);

WDD<-WDD[,xx]

}

## vif Check on training data

if (length(xx)>1) {

vifChk<-vifSelect(WDD[rowTrain,],t=vif.CUT,fix=NA)

xx<-xx[vifChk[[1]]]; #remove VIF

w0<-c(w0,"<p>Check collinearity (VIF stepwise selection) on training data:", mat2htmltable(vifChk[[3]]), "</p>")

if (length(vifChk[[2]])>0) w0<-c(w0,"<p>Variables removed: ", paste(vifChk[[2]],collapse=" "),"</p>")

}

## predictors left

xx<-sort(xx)

w0<-c(w0,"<p><strong>Predictors used:</strong>", paste(xx,collapse=" "),"</p>")

WDD<-WDD[,xx]

td<-(rowTrain & !is.na(WDY))

vd<-(!rowTrain & !is.na(WDY))

##features plot

png(paste(ofname,"_xy_box.png",sep=""))

featurePlot(x=WDD[td,], y=WDY[td], plot="box",strip=strip.custom(par.strip.text=list(cex=.7)))

dev.off()

png(paste(ofname,"_xy_density.png",sep=""))

featurePlot(x=WDD[td,], y=WDY[td], plot="density",strip=strip.custom(par.strip.text=list(cex=.7)))

dev.off()

orig<-cbind(WDY,WDD); colnames(orig)[1]<-colvname

## check MFP

chkmfp<- (sum(cbl==2)>0);

xx.cont<-xx[!is.na(match(xx,xxv[xxl==0]))]

xx.mfp<-NULL; xx.mfp1<-NULL; xx.mfp2<-NULL; mfp.newname<-NULL; mfp.origname<-NULL; mfp.formula<-NULL

if (chkmfp & length(xx.cont)>0) {

w0<-c(w0,"<p><strong>Check MFP for continuous variables:</strong>")

tmpd<-data.frame(cbind(1*(WDY==1L),WDD[,xx.cont]))

names(tmpd)<-c(colvname,xx.cont)

xx0<-paste("fp(",xx.cont,")",sep="")

fmlp<-paste(colvname,"~",paste(xx0,collapse="+"))

xfp<-try(mfp(formula(fmlp),family=binomial(link="logit"),select=0.05,alpha=0.05,data=tmpd))

rm(tmpd)

xx.mfp<-rownames(xfp$fptable)[xfp$fptable[,"df.final"]==2 | xfp$fptable[,"df.final"]==4]

xx.mfplist<-list(); tmpx<-NULL; tmpsxf<-NULL;

for (x in xx.mfp) {

xx.mfplist[[x]]<-c()

tmp<-unlist(strsplit(xfp$trafo[x,], "\\)\\+I\\("))

if (length(tmp)==1) xx.mfplist[[x]]<-tmp[1]

if (length(tmp)==2) xx.mfplist[[x]]<-c(paste(tmp[1],")",sep=""), paste("I(",tmp[2],sep=""))

if (length(xx.mfplist[[x]])>0) {

xx.mfp1<-c(xx.mfp1, x)

tmpx<-cbind(tmpx, eval(parse(text = str_replace_all(xx.mfplist[[x]][1],x,paste("WDD[,'",x,"']",sep="")))))

mfp.newname<-c(mfp.newname, paste(x,"1",sep="."));

mfp.origname<-c(mfp.origname, x);

mfp.formula<-c(mfp.formula, gsub("I\\(", "\\(", xx.mfplist[[x]][1]))

tmpsxf<- c(tmpsxf, sxf[which(xvname==x)])

xx<-xx[-which(xx==x)];

}

if (length(xx.mfplist[[x]])>1) {

xx.mfp2<-c(xx.mfp2, x)

tmpx<-cbind(tmpx, eval(parse(text = str_replace_all(xx.mfplist[[x]][2],x,paste("WDD[,'",x,"']",sep="")))))

mfp.newname<-c(mfp.newname, paste(x,"2",sep="."));

mfp.origname<-c(mfp.origname, x)

mfp.formula<-c(mfp.formula, gsub("I\\(", "\\(", xx.mfplist[[x]][2]))

tmpsxf<- c(tmpsxf, sxf[which(xvname==x)])

}

}

if (!is.null(tmpx)) {

tmpcname<-c(colnames(WDD),mfp.newname) ; WDD<-cbind(WDD,tmpx); colnames(WDD)<-tmpcname;

xxv<-c(xxv,mfp.newname); xxl<-c(xxl,rep(0,length(mfp.newname))); xxf<-c(xxf, tmpsxf);

xx<-c(xx,mfp.newname)

WDD<-WDD[,xx]

tmpcname<-c(colnames(orig),mfp.newname); orig<-cbind(orig,round(tmpx,6)); colnames(orig)<-tmpcname

}

if (length(xx.mfp)>0) {

tmp<-c("MFP variable","Formula")

for (x in xx.mfp) {

tmp<-rbind(tmp, c(paste(x,".1",sep=""), xx.mfplist[[x]][1]))

if (length(xx.mfplist[[x]])>1) tmp<-rbind(tmp, c(paste(x,".2",sep=""), xx.mfplist[[x]][2]))

}

w0<-c(w0,mat2htmltable(tmp),"</p>")

} else {

w0<-c(w0,"None multivariable fractional polynomials found</p>")

}

}

## setup models

nxx<-length(xx); xx.xlv<-rep(0,nxx); xx.xvname<-rep("",nxx); xx.xb<-rep("",nxx); tmpsxf<-c(); xx.sxf<-rep(0,nxx);

fndError<-FALSE; m<-c(); tmpm.xx<-list()

for (i in 1:nxx) {

for (j in 1:length(xxv)) {

if (xxl[j]==0 && xxv[j]==xx[i]) {

xx.xvname[i]<-xxv[j]; xx.xlv[i]<-0; xx.sxf[i]<-1*(xxf[j]!="0");

tmpsxf<-unlist(strsplit(paste(xxf[j]),",")); xx.xb[i]<-xb[j]

break;

}

if (xxl[j]>0) {

tmpl<-nchar(xxv[j])+1

if (substr(xx[i],1,tmpl)==paste(xxv[j],".",sep="") & nchar(xx[i])>tmpl) {

xx.xvname[i]<-xxv[j]; xx.xlv[i]<-2; xx.sxf[i]<-1*(xxf[j]!="0");

tmpsxf<-unlist(strsplit(paste(xxf[j]),","));

xx.xb[i]<-paste(xb[j],"=", substr(xx[i],tmpl,99))

break;

}

}

}

if (xx.xvname[i]=="") {fndError<-TRUE; break;}

for (j in tmpsxf) {if (j %in% m) {tmpm.xx[[trim(j)]]<- sort(c(tmpm.xx[[trim(j)]], xx[i]));} else {tmpm.xx[[trim(j)]]<-xx[i];}}

m<-sort(unique(c(m,tmpsxf)))

}

if (length(m)==0 | fndError) {m<-c(0); tmpm.xx<-list(); tmpm.xx[["0"]]<-sort(xx);}

xx.rfe<-NULL

##recursive features selection

if (sum(cbl==1)>0 & ncol(WDD)<20) {

ctrl<-rfeControl(functions=rfFuncs,method="repeatedcv",repeats=5,verbose=FALSE)

rfeResult<-rfe(x=WDD[td,xx], y=WDY[td], sizes=c(2:ncol(WDD)), rfeControl=ctrl)

xx.rfe<-sort(rfeResult$optVariables)

w0<-c(w0,"<p>Feature selection using recursive feature elimination (RFE):")

w0<-c(w0,mat2htmltable(rbind(names(rfeResult$results),numfmt(rfeResult$results,5)),0,NA),"</p>")

w0<-c(w0,"<p><strong>RFE Selected variables:</strong>",paste(xx.rfe,collapse=" "),"</p>")

}

if (!is.null(xx.rfe)) {for (i in m) {if (identical(tmpm.xx[[trim(i)]],sort(xx.rfe))) {xx.rfe<-NULL; break;}}}

chkintr<- (sum(cbl==3)>0);

m.xx<-list();

if (chkintr & !is.null(xx.rfe)) {

m.xx[[1]]<-xx.rfe;

} else {

if (!is.null(xx.rfe)) {m<-c(m,"9"); tmpm.xx[["9"]]<-xx.rfe;}

if (m[1]=="0" && length(m)>1) m<-m[-1]

m.xx[[1]]<-tmpm.xx[[trim(m[1])]]

if (length(m)>1) {

for (i in m[-1]) {

k<-length(m.xx); fnd<-FALSE

for (j in 1:k) {if (identical(tmpm.xx[[trim(i)]], m.xx[[j]])) {fnd<-TRUE; break;}}

if (!fnd) m.xx[[k+1]]<-tmpm.xx[[trim(i)]]

}

}

}

## add interactions

m.sxf1<-xx.sxf[match(m.xx[[1]],xx)]

if (chkintr & length(m.xx)==1 & sum(m.sxf1)>0) {

f <- as.formula(WDY ~ .*.);

WDD1 <- model.matrix.lm(f, data.frame(cbind(WDY,WDD[,m.xx[[1]]])), na.action="na.pass")[,-1]

if (!is.null(mfp.newname) & length(unique(mfp.origname))<length(mfp.newname)) {

tmpname<- strsplit(colnames(WDD1),":")

WDD1<-WDD1[, unlist(lapply(tmpname,function(x) {

if (length(x)!=2) return(TRUE);

tmp<-match(x,mfp.newname); tmp<-tmp[!is.na(tmp)]; if (length(tmp)!=2) return(TRUE)

return(length(unique(mfp.origname[tmp]))==2)

}))]

}

if (sum(m.sxf1==1)<length(m.xx[[1]])) {

tmpname<- strsplit(colnames(WDD1),":")

tmpex<-m.xx[[1]][m.sxf1!=1];

tmpexi<-unlist(lapply(tmpname, function(x) return(length(x)==2 & sum(!is.na(match(x,tmpex)))>0)))

WDD1<-WDD1[,!tmpexi]

}

set.seed(222)

y<-1*(WDY==1L);

cv.mdl<-cv.glmnet(WDD1[td,],y[td],family="binomial",nfold=10,type.measure="auc")

xname <- rownames(coef(cv.mdl))

beta.1se <- round(as.vector(coef(cv.mdl,s="lambda.1se")),6)

xslt.1se <- xname[beta.1se !=0 & xname!="(Intercept)"]

beta.min <- round(as.vector(coef(cv.mdl,s="lambda.min")),6)

xslt.min <- xname[beta.min !=0 & xname!="(Intercept)"]

coef.tmp <- rbind(c("","lambda.1se", "lambda.min"),cbind(xname, beta.1se, beta.min))

m.xx[[1]]<-unique(c(unlist(strsplit(xslt.min,":")),xslt.min))

allxx<-sort(m.xx[[1]])

w0<-c(w0,"<p><strong>LASSO selection (based on AUC) for interaction terms:</strong>")

w0<-c(w0,mat2htmltable(coef.tmp),"</p>")

w0<-c(w0,"<p><strong>Selected variables:</strong>",paste(m.xx[[1]],collapse=" "),"</p>")

tmp<-is.na(match(xx,colnames(WDD1)))

if (sum(tmp)>0) {tmp1<-cbind(WDD[,xx[tmp]]);colnames(tmp1)<-xx[tmp]; WDD<-cbind(tmp1,WDD1);rm(tmp1);} else {WDD<-WDD1;}

rm(WDD1, tmp)

}

rm(tmpm.xx)

nmdl<-length(m.xx); m.lb<-paste("Model",1:nmdl); if (!is.null(xx.rfe)) m.lb[1]<-"RFE selected";

## train

if (sum(cbl==4)>0) {trControl<-trainControl(method='repeatedcv',number=10,repeats=5)

} else if (sum(cbl==5)>0) {trControl<-trainControl(method='boot',number=100)

} else {trControl<-trainControl(method='none')

}

mdlSummaryROC<-function(xv=c(),set=td, tv="T", note="training sample", m="") {

if (m>"") note<-paste(note, " (model", m, ")",sep="")

cft<-confusionMatrix(predict(mdl,WDD[set,xv],'raw'), WDY[set], positive="1")

tmp<-numfmt(cft$overall[c("Accuracy","AccuracyLower","AccuracyUpper","AccuracyNull","AccuracyPValue")],3);

rr<-paste("Predict",rownames(cft$table),sep=": ")

rw<-paste("Ref (0,1): (", cft$table[1,1],", ",cft$table[1,2],")",sep="")

rw<-c(rw,paste("Ref (0,1): (",cft$table[2,1],", ",cft$table[2,2],")",sep=""))

rr<-c(rr,"Accuracy (95%CI)","Accuracy Null","P-value (Accuracy > Null)")

rw<-c(rw,paste(tmp[1]," (",tmp[2],", ",tmp[3],")",sep=""),tmp[4],tmp[5])

rr<-c(rr,names(cft$byClass))

rw<-c(rw,numfmt(cft$byClass,4))

predT<-predict(mdl,newdata=WDD[set,xv],type="prob")[,2]

prT <- prediction(predT,WDY[set])

perf<- performance(prT, measure = "tpr",x.measure = "fpr")

WDY.set<-as.numeric(levels(WDY[set]))[as.numeric(WDY[set])]

aucT<-auc(WDY.set,predT)

mainb<-paste("ROC for",note,"\nAUC =", round(aucT,3))

png(paste(ofname,m,"_ROC_",tv,".png",sep="")); plot(perf, main=mainb); dev.off();

pdf(paste(ofname,m,"_ROC_",tv,".pdf",sep=""),width=pdfwd, height=pdfht,family="GB1"); plot(perf,main=mainb); dev.off();

calibratePlot(WDY[set],predT,paste(ofname,m,"_cal_",tv,sep=""),mainb=paste("Calibration for",note))

rc<-cbind(unlist(prT@cutoffs),unlist(prT@tp),unlist(prT@fp),unlist(prT@fn),unlist(prT@tn))

rc<-cbind(rc,round(unlist(perf@x.values),5), round(unlist(perf@y.values),5))

colnames(rc)<-c("Cutoff","True-positive","False-positive","False-negative","True-negative","Sensitivity","1-specificity")

outroct<-paste(ofname,m,"_ROC_T.xls",sep="")

write.table(rc,file=paste(ofname,m,"_ROC_",tv,".xls",sep=""),row.names=FALSE,col.names=TRUE,sep="\t",append=FALSE,quote=FALSE)

rr<-c(rr,"Area under curve (AUC)")

rw<-c(rw,numfmt(aucT, 4))

tmpd<-cbind(WDY.set, predT); colnames(tmpd)<-c(colvname, "Predict.prob")

tdca<-dca(formula(paste(colvname,"~Predict.prob")),data=data.frame(tmpd),thresholds=seq(0,0.99, by=0.01))

pp<-plot(tdca,smooth=TRUE)

png(paste(ofname,m,"_dca_",tv,".png",sep="")); print(pp); dev.off();

pdf(paste(ofname,m,"_dca_",tv,".pdf",sep=""),width=pdfwd, height=pdfht,family="GB1"); print(pp); dev.off();

outdcat<-paste(ofname,m,"_dca_T.xls",sep="")

aa<-tdca$dca

ra<-cbind(as.character(aa$label),round(aa$threshold,2),round(aa$tp_rate,4),round(aa$fp_rate,4))

ra<-cbind(ra,round(aa$net_benefit,4),aa$harm,aa$n,round(aa$prevalence,4))

colnames(ra)<-c("label","threshold","tp_rate","fp_rate","net_benefit","harm","n","prevalence")

return(list(unlist(perf@x.values), unlist(perf@y.values), aucT, rr, rw, ra))

}

rr<-list(); rb<-c(); k<-0; rname<-c(); rcc<-NULL; rdcaT<-NULL; rdcaV<-NULL

acckappa<-c("Accuracy ± SD", "Kappa ± SD")

noteb<-c("training","validation"); if (sum(!rowTrain)==0) noteb<-c("","")

dcatb<-c("Model","Train","label","threshold","tp_rate","fp_rate","net_benefit","harm","n","prevalence")

nb<-NULL; pred.prob1<-NULL;

for (m in 1:nmdl) {

xxi<-m.xx[[m]];

x<-WDD[td,xxi]; if (length(xxi)==1) {x<-matrix(x,ncol=1);colnames(x)<-xxi;}

mdl<-train(x,y=WDY[td],method='glm',family='binomial',trControl=trControl,metric="Accuracy")

if (m==1) {

v.imp<-varImp(mdl)$importance; v.imp<-cbind(rownames(v.imp),v.imp)

v.imp<-v.imp[order(v.imp[,2],decreasing=TRUE),]; v.imp[,2]<-numfmt(v.imp[,2],2)

w0<-c(w0,"<p>Variable importance:",mat2htmltable(v.imp),"</p>")

}

pred.prob1<-cbind(pred.prob1, round(predict(mdl,newdata=WDD[,xxi],type="prob")[,2],4))

acc<-paste(numfmt(mdl$results["Accuracy"],4), " ± ", numfmt(mdl$results["AccuracySD"],4),sep="")

kap<-paste(numfmt(mdl$results["Kappa"],4), " ± ", numfmt(mdl$results["KappaSD"],4),sep="")

acckappa<-cbind(acckappa, c(acc,kap))

smdl<-summary(mdl$finalModel)

coe<-smdl$coefficients

oo<-numfmt(exp(cbind(coe[,1], coe[,1]-1.96*coe[,2], coe[,1]+1.96*coe[,2])),2)

oo<-cbind(numfmt(coe[,-4],4),oo,pvformat(coe[,4],4))

oo<-rbind(c(colnames(coe)[-4],"OR","OR.lower","OR.upper","P-value"),oo)

oo<-cbind(c("",rownames(coe)),oo)

os<-paste(numfmt(smdl$null.deviance,4), " (df = ", smdl$df.null,")",sep="")

os<-c(os, paste(numfmt(smdl$deviance,4), " (df = ", smdl$df.residual,")",sep=""))

os<-c(os, numfmt(smdl$aic,2), numfmt(logLik(mdl$finalModel),4))

os<-cbind(c("Null deviance","Residual deviance","AIC","Log Likelihood"),os)

finalFML<-coe2formula(smdl$coefficients)

w0<-c(w0, "<p><strong>Models output</strong>")

w0<-c(w0, "<p><strong>", m.lb[m], ":</strong> ",paste(xxi,collapse=", "),"</p>")

w0<-c(w0,"<p>Model equation:</p><p>",finalFML,"</p><p>",mat2htmltable(oo,0,NA),"</p>")

w0<-c(w0,"<p>",mat2htmltable(os,0,NA),"</p>")

k<-k+1

if (nmdl==1) {mp<-"";} else {mp<-paste("_",m,sep="");}

rr[[k]]<-mdlSummaryROC(xv=xxi, set=td, tv="T", note="training sample", m=mp)

rb<-c(rb, paste(m.lb[m], noteb[1]))

if (m==1) {rname<-rr[[k]][[4]]; rcc<-cbind(rname,rr[[k]][[5]]);

} else {rcc<-cbind(rcc, rr[[k]][[5]][match(rname, rr[[k]][[4]])]);

}

dcatb<-rbind(dcatb, cbind(m,1,rr[[k]][[6]]))

nb<-cbind(nb, as.numeric(rr[[k]][[6]][,5]))

if (sum(!rowTrain)>0) {

k<-k+1

rr[[k]]<-mdlSummaryROC(xv=xxi, set=vd, tv="V", note="validation sample", m=mp)

rb<-c(rb, paste(m.lb[m], noteb[2]))

rcc<-cbind(rcc, rr[[k]][[5]][match(rname, rr[[k]][[4]])]);

if (m==1) {rdcaV<-rr[[k]][[6]][,c(1,2,5)];} else {rdcaV<-cbind(rdcaV, rr[[k]][[6]][,5]);}

dcatb<-rbind(dcatb, cbind(m,0,rr[[k]][[6]]))

nb<-cbind(nb, as.numeric(rr[[k]][[6]][,5]))

}

}

colnames(pred.prob1)<-paste("pred.model",1:m,sep="")

orig<-cbind(orig,pred.prob1)

orig[,1]<-orig[,1]-1

orig<-cbind(td*1, orig); colnames(orig)[1]<-"_train_";

write.table(orig,file=paste(ofname,"_pred.xls",sep=""),row.names=FALSE,col.names=TRUE,sep="\t",append=FALSE,quote=FALSE)

if (k>1) {

png(paste(ofname,"_ROC.png",sep="")); mrocplot(rr, roclb=rb); dev.off()

pdf(paste(ofname,"_ROC.pdf",sep=""),width=pdfwd, height=pdfht,family="GB1"); mrocplot(rr, roclb=rb); dev.off()

rcc<-rbind(c("",rb), rcc)

}

acckappa<-rbind(c("", rb), acckappa)

write.table(dcatb,file=paste(ofname,"_dca.xls",sep=""),row.names=FALSE,col.names=TRUE,sep="\t",append=FALSE,quote=FALSE)

th<-seq(0,0.99, by=0.01)

yupp<-ceil(max(nb[1,])*100)/100

ylow<-floor(min(nb[min(which(th>min(nb[1,]))),])*100)/100

doPlot<-function(byModel=TRUE, tv=0, nc=2) {

plot(x=c(0,1),y=c(0,0), type="l", lty=1, xlim=c(0,1),ylim=c(ylow,yupp),xlab="Threshold probability", ylab="Net benefit")

par(new=TRUE)

if (byModel) {

ycut <- min(which(is.na(c(nb[1:100,tv+1],NA))))-1; lines(th[1:ycut],nb[1:ycut,tv+1], lty=2, lwd=1)

for (i in 1:nmdl) {

icol<-nc*(i-1)+1+tv

ycut <- min(which(is.na(c(nb[201:300,icol],NA))))-1

lines(th[1:ycut], nb[200+(1:ycut),icol], lty=2+i, lwd=2)

}

legend(x="topright", legend=c("Treat none","Treat all",m.lb), lty=c(1,2,2+(1:nmdl)),lwd=c(1,1,rep(2,nmdl)))

} else {

ycut <- min(which(is.na(c(nb[1:100,1],NA))))-1; lines(th[1:ycut], nb[1:ycut,1], lty=2, lwd=1);

ycut <- min(which(is.na(c(nb[201:300,1],NA))))-1; lines(th[1:ycut], nb[200+(1:ycut),1], lty=3, lwd=2)

ycut <- min(which(is.na(c(nb[1:100,2],NA))))-1; lines(th[1:ycut], nb[1:ycut,2], lty=4, lwd=1)

ycut <- min(which(is.na(c(nb[201:300,2],NA))))-1; lines(th[1:ycut], nb[200+(1:ycut),2], lty=5, lwd=2)

legend(x="topright",legend=c("Treat none","Treat all for TD","Model for TD","Treat all for VD", "Model for VD"),lty=(1:5),lwd=c(1,1,2,1,2))

}

par(new=FALSE)

}

if (nmdl>1) {

if (sum(!rowTrain)>0) {

png(paste(ofname,"_dca_T.png",sep="")); doPlot(byModel=TRUE,tv=0,nc=2); dev.off()

pdf(paste(ofname,"_dca_T.pdf",sep=""),width=pdfwd, height=pdfht,family="GB1"); doPlot(byModel=TRUE,tv=0,nc=2); dev.off()

png(paste(ofname,"_dca_V.png",sep="")); doPlot(byModel=TRUE,tv=1,nc=2); dev.off()

pdf(paste(ofname,"_dca_V.pdf",sep=""),width=pdfwd, height=pdfht,family="GB1"); doPlot(byModel=TRUE,tv=1,nc=2); dev.off()

} else {

png(paste(ofname,"_dca.png",sep="")); doPlot(byModel=TRUE,tv=0,nc=1); dev.off()

pdf(paste(ofname,"_dca.pdf",sep=""),width=pdfwd, height=pdfht,family="GB1"); doPlot(byModel=TRUE,tv=0,nc=1); dev.off()

}

} else if (sum(!rowTrain)>0) {

png(paste(ofname,"_dca.png",sep="")); doPlot(byModel=FALSE); dev.off()

pdf(paste(ofname,"_dca.pdf",sep=""),width=pdfwd, height=pdfht,family="GB1"); doPlot(byModel=FALSE); dev.off()

}

w0<-c(w0,"<p>",mat2htmltable(rcc),"</p>")

w0<-c(w0,"<p>Internal validation:",mat2htmltable(acckappa),"</p>")

w<-c("<!DOCTYPE html><html lang='zh'><head><meta charset='utf-8'></head><body>")

w<-c(w,"<h2>",title,"</h2>")

w<-c(w,"</br>Outcome:", yb, "</br></br>")

w<-c(w,w0,"</br>")

w<-c(w,wd.subset)

w<-c(w,paste("</br></br>Created by EmpowerStats (www.empowerstats.com) and R on",Sys.Date()))

w<-c(w,"</body></html>")

fileConn<-file(paste(ofname,".htm",sep="")); writeLines(w, fileConn)
